# Supplementary material for: Flow control of three-dimensional cylinders transitioning to turbulence via multi-agent reinforcement learning
Source: Commun Eng. 2025 Jun 18;4:113. doi: 10.1038/s44172-025-00446-x (PMC12177081; doi:10.1038/s44172-025-00446-x)
Supplement: Supplementary file 1 — Supplementary Information [file 44172_2025_446_MOESM1_ESM.pdf]

This file contains all reviewer reports in order by version, followed by all author rebuttals in order by version.

nature portfolio

Peer Review File

Flow control of three-dimensional cylinders transitioning to turbulence via multi-agent reinforcement learning

Corresponding Author: Mr Pol Suárez

Version 0:

Reviewer comments:

Reviewer #1

(Remarks to the Author)
The authors applied the reinforcement learning guided active control for flow past a circular cylinder in the transitional regime. The 3D simulation environment is applied in the training. The whole manuscript is very interesting and is written in an understandable manner. From my view, this work deserves to be considered to be published in Communications Engineering, once the following problems are addressed.
My major concerns are twofold:
First, the numerical results are not validated. The mesh convergence study is also not performed. Flow in the transitional regime is sensitive to the computational scheme, the mesh resolution, the spanwise length, etc. This problem needed to be thoroughly addressed to convince the readers.
Second, the manuscript lacks some essential discussions concerning the physical mechanism. The physical rationale behind the successful control relied much on the flow stability, especially the two distinct modes of 3D instability, which is also mentioned in the introduction part. However, the later discussions only focus on the instantaneous flow fields and statistical data, which are not enough to support any conclusion related to 3D stability.
Specifically, I also have the following minor concerns.
1.In Figure 1, the periodic control is applied. It is questionable how these open-loop controls are found? Are they optimal? More details needed to be provided.
2. Figure 1(a) seems to be not well converged.
3. Eq.1 is used to quantify the flow rate of jet actuators, which practice is indeed common as a penalization term. However, can the authors also consider to quantify the energy consumption?
4. The spectra in Figure 2(b) are not well produced. The high-frequency region needs to be supplemented.
5. Figure 5(d, e) shows the velocity deficit, can the profiles be further related to drag reduction?
6. In Page 23, a table that lists all the hyper-parameters is recommended.

Reviewer #2

(Remarks to the Author)
This paper utilize multi-agent reinforcement learning (MARL) to design active flow control strategies for the 3D flow around the cylinder. The flow control is considering multiple zero-net-mass-flux jet on top of the cylinder with the Reynolds number ranging from 100 to 400 covering the transition to 3D wake instabilities. This work is interesting and well-written, and it demonstrate that MARL can effective design an active flow control to achieve drag reduction and also show performance better than classical periodic control. Some comments below may help improve:

1. In figure 4, although the DRL is able to reduce the drag coefficient, the amplitude of lift coefficient is also reduced. This may be unfavorable in aerospace applications. Would the authors discuss this trade-off between drag coefficient and lift coefficient?

2. Moreover, this work compared their flow control results with periodic control. However, how does this compared with other recent deep reinforcement learning results is suggested to be discussed. Is the authors using essentially the same reinforcement learning framework as others use, such as Ref [29], or the MARL in this paper has its own advantage?

3. Page 23 the authors mentioned 'This algorithm demonstrates robustness, as it is somewhat forgiving with hyperparameter initializations'. I would be interested in seeing a robustness study regarding hyperparameter initialization.

4. It is also suggested to discuss what is the challenge to apply this to higher Reynolds number turbulent flows.

Several Typos:

a. On the top of page 5, 'The transion range of for 2D ... '

b. Caption of figure 2, '(b) Power-spectral density of the streamwise velocity u evaluated at at the streamwise location... '

c. Page 21, caption of figure 6, 'Note that is a schematic representation...' looks to miss the subject in this sentence

d. Page 22, 'Drag and lift forces (Fx and Fy, respectively) forces are obtained ...'.

Reviewer #3

(Remarks to the Author)
The article presents the application of Deep Reinforcement Learning (DRL) to the flow around a circular cylinder at low Reynolds number and demonstrates that it can be used to reduce drag, via incorporation with an active flow control (AFC) mechanism. The work is ambitious and likely to be novel in a number of aspects. In the first case, although the Reynolds number range is low, it includes instances where the wake flow is three dimensional. Correspondingly, the AFC employed varies in the span wise direction. A considerable number of simulations were undertaken to train the model before the resulting framework was applied in an exploitation mode. In general I am in favour of this work being published, as it provides evidence for what should become an important branch of flow control in the coming years. However, there are a few issues to be discussed further, as noted below:

Conceptual
- One of the main questions at present is whether or not the benefit of the multi agent vs single agent approach is fully demonstrated, ie MARL vs SARL. This is important as it is one of the main objectives and stated novelties of the work.
- Figure 3 provides an interesting view of the resulting mass flow across the jets from the exploitation phase of the DRL calculation. For Re=100 and 200, as stated by the authors, the flow is 2D and as such the resulting jet mass flow pattern is constant in spanwise as expected, but this raises two questions; 1) Is there a fundamental reason why the periodic control algorithm wis not able to achieve the same performance? 2) would SARL have been able to obtain the same result and if so would it have been cheaper?
- For Re=300 and 400 in fig 3, the variation of spanwise mass flow rate is shown, demonstrating the potential of the MARL via the independent actuation of the ten jets. However there are two further questions here: 1) there is a clear underlying frequency in both cases – how would a periodic control (PC) based on this frequency alone perform? Presumably SARL would be able to capture this frequency. 2) as such, in order to evaluate the net benefit of MARL over SARL, perhaps there should be a test of the significance of the spanwise variation over this baseline frequency. To what extent are DRL prescribed variations away from this frequency beneficial to the overall flow control?
- The plots of Q with time in Figure 4a) and b) demonstrate the variation with time of mass flow rate. It is clear that there is relatively low spanwise variation of the jet mass flow, particularly once the 'transition' or adjustment period has been completed. Would a single jet (i.e. presumbably via SARL) have obtained the same variation as that which is shown to be the predominant variation of Q? Did the authors explore the sensitivity of the jet width to the overall solution? E.g. starting with two jets per span and gradually increasing.
- Some of the conclusions made towards the end of the study, e.g. p19, first line, are around the performance of DRL vs PC strategies. Are authors confident, to a reasonable degree at least, that there isn't a PC strategy that can achieve similar overall performance to the obtained DRL solution?

Flow Physics
- At present, the emphasis of this work is placed more on the fact that the AFC is possible via the DRL framework employed, rather than a discussion of the flow physics of the identified DRL solution. Perhaps the latter could be explored further, particularly where it is relevant to the overall concept being demonstrated; i.e. that DRL results in non-intuitive solutions to be developed. This is important to the conclusion since as noted below there is only relatively minor spanwise variation of Q.
- Could authors discuss the plateau observed in Cp for Re=400, as per figure 5f, at around theta=-90 degrees.
- Could the authors provide indication of the boundary layer resolution and perhaps provide further insight on the impact of AFC on the location of boundary layer separation. This could be achieved via the analysis of velocity profiles in this region, and/or perhaps more succinctly via plotting of skin friction coefficient.
- As observed in Fig 4, there is an absence of unsteady flow structures in the immediate wake of the cylinder when the control is active; particularly in the region around 1 radius downstream of the aft of the cylinder. Could this be explored and explained in further detail?

Aspects of the DRL algorithm
- What is the sensitivity of the algorithm to the location of observation states S_i? How does the location of the three slices arranged with respect to each individual jet – are they contained within the same spanwise extent? It is not clear.
- What is the sensitivity to the quantity \Beta introduced at the top of p25? It appears that this quantity relates to the overall ability of the MARL to incorporate information from the non-local spanwise position. This would therefore appear to be a relevant parameter to explore in the context of the overall objectives and conclusions.
- Can authors expand on the statements made on the comments regarding the limitation placed on the maximum values of Q in the algorithm employed. Specifically; p13 (4 lines from btm) 'less than 10% of the maximum possible value'; and p24 'Q_max=0.176 was set based on our experience…'. Why where these limitations necessary? Would it not be beneficial in

some instances to permit larger active control?

Numerical considerations
- We presume that the code has been suitably validated for these flows, but it would be useful to confirm as such. It would also be helpful for repeatability to provide some details of spatial resolution employed, e.g. an image of mesh resolution to demonstrate where refinement has been prioritised.
- Consideration of figure 1b gives the impression that in some cases the flow has not fully developed, particularly for Re=300 and 400. Are authors confident that the DRL performance would remain similar for all stages of the flow. It is known from the literature that lower frequencies start to become more prominent – would the DRL be able to discern and adjust to counter these frequencies?
- Some discrete behaviour in the solution is observed for the uncontrolled case in fig 5e e.g. x/D=9. Could the origin of this be explained?

Typos/Minor:
p3 top: irruption -> eruption
p3 ‘table top games’ -> ‘board games’?
p15, fig 4. The line plots have x-axis label tU/D, while titles above flow vis. Frames have just t. Perhaps they should instead be t*=tU/D, or similar.
P20: Are the two quantities ||U(Q, theta)|| in eqn (2) and ||U_jet||, defined three lines later, the same quantity? If so this should be clarified.

Version 1:

Reviewer comments:

Reviewer #1

(Remarks to the Author)
The authors have thoughtfully addressed all concerns raised during the review process. With these revisions, the manuscript now meets the journal’s standards for rigor and clarity. I am pleased to recommend acceptance of the current version.

Reviewer #2

(Remarks to the Author)
The authors have addressed my comments and improved their manuscript. I would like to suggest the publication of this paper.

Reviewer #3

(Remarks to the Author)
I have read through the responses and updates and I thank the authors for their comprehensive revision. Amongst other changes, the additional information provided in this version regarding code validation, sensitivity of key parameters and the new flow physics study has fully addressed the queries raised in the last review. I recommend this work for publication.

Alistair Revell

Open Access This Peer Review File is licensed under a Creative Commons Attribution 4.0 International License, which permits use, sharing, adaptation, distribution and reproduction in any medium or format, as long as you give appropriate credit to the original author(s) and the source, provide a link to the Creative Commons license, and indicate if changes were made.
In cases where reviewers are anonymous, credit should be given to 'Anonymous Referee' and the source.
The images or other third party material in this Peer Review File are included in the article's Creative Commons license, unless indicated otherwise in a credit line to the material. If material is not included in the article's Creative Commons license and your intended use is not permitted by statutory regulation or exceeds the permitted use, you will need to obtain permission directly from the copyright holder.
To view a copy of this license, visit https://creativecommons.org/licenses/by/4.0/

We appreciate the thoughtful feedback from the reviewer, which has helped us to improve our work. In this rebuttal, we address the concerns raised, clarify ambiguities, and outline the steps taken to strengthen the manuscript. Our goal is to ensure that the revised paper meets standards of the journal and resolves all issues raised.
Each question is addressed in red, and any necessary changes to the manuscript are incorporated in blue.

Reviewer #1 (Remarks to the Author):

The authors applied the reinforcement learning guided active control for flow past a circular cylinder in the transitional regime. The 3D simulation environment is applied in the training. The whole manuscript is very interesting and is written in an understandable manner. From my view, this work deserves to be considered to be published in Communications Engineering, once the following problems are addressed.

My major concerns are twofold:

1. First, the numerical results are not validated. The mesh convergence study is also not performed. Flow in the transitional regime is sensitive to the computational scheme, the mesh resolution, the spanwise length, etc. This problem needed to be thoroughly addressed to convince the readers.

The problem has been properly addressed by adding Appendix 1, which includes prior grid independence studies from an earlier phase that were not part of the previous manuscript. In the appendix we report Re_D = 400 case as a reference. The quantities of interest were the convergence of C_d and RMS of C_d and C_l over time, which are in good agreement with the values reported in the literature. Note that grid independence was also achieved for all Re_D cases, and the chosen mesh was M1 (medium), which offers a good balance between computational cost and accurately capturing the physics of the problem. This balance is crucial for any DRL training setup, given the high computational cost already associated with it. In the same Appendix 1, the used parametrized mesh is also described. Additionally, a comment in the results section referencing Appendix 1 has been added.

To further validate the findings, a column has been added to the table in Figure 2(c) to compare the Strouhal number with the literature, also in very good agreement. In this case, the shedding modes are also identified.

Changes in the manuscript

In Results section:

We then evaluate the optimal model and compare it with the uncontrolled cases and the periodic control (PC). Note that the uncontrolled converged results across Re_D are obtained after a grid-independence study—more details can be found in Appendix 1. For the study of the periodic control, more details are also provided in Appendix 2, where the tables and heatmaps from the optimization problem are presented. These results will be used for the subsequent analysis. In doing so, we investigate the utilization of the trained DRL agents for exploitation without involving any

exploration. Statistical analyses are conducted to elucidate the underlying physical mechanisms responsible for drag reduction, leading to potential energy savings.

Appendix 1 added:

A grid-independence study was conducted to select an optimal mesh that balances low computational cost and accurate physical representation. This step is particularly important when working with the DRL framework due to its high computational demands and the necessity of parallelizing multiple simulations simultaneously.
The study involves iterating over a parametrized mesh, adjusting specific values to control the resolution in the \(xy\)-plane and the number of uniformly spaced elements along the \(z\)-axis. Note that lengths $L_x/D=30$ and $L_y/D=15$ are the same for all cases. The schematic illustrates the placement of these elements and the parameters that can be adjusted. The table lists the details of each mesh—ranging from coarse to finer resolutions—and the resulting statistics for the quantities of interest, such as \(\overline{C_D}\), \(\overline{C_{D_{\rm RMS}}}\), and \(\overline{C_{L_{\rm RMS}}}\), calculated over the entire surface. To ensure temporal convergence, 200 time units were found to be more than sufficient to achieve it for this Re_D range.

The results obtained for the drag coefficient (\(C_D\)) of a circular cylinder in the Reynolds-number range between 100 to 400—\(C_D = 1.4\) at \(Re_D = 100\), \(1.38\) at \(Re_D = 200\), \(1.3\) at \(Re_D = 300\), and \(1.21\) at \(Re_D = 400\)—are in good agreement with the ranges reported in the literature (\(C_D \approx 1.2 - 1.4\) at \(Re_D = 100\), \(1.1 - 1.3\) at \(Re_D = 200\), \(1.0 - 1.2\) at \(Re_D = 300\), and \(1.0 - 1.2\) at \(Re_D = 400\)). Some scatter in the data is observed, which can be attributed to the coexistence of two modes in this transition regime, as discussed in the works of \cite{tritton_1959, schlichting2016boundary, white2006viscous}. Regarding the Strouhal number St, the literature exhibits less scattering across \(Re_D\) and better alignment with the reported values in the experimental and numerical literature. The first columns in the table in Figure~\ref{fig:fig2}(c) illustrate this agreement, comparing \(St\) values from the literature (Williamson, 1996 \cite{williamson_vortex_1996}) with the present statistics. Furthermore, the \(St\) values enable us to associate modes with our results as well.

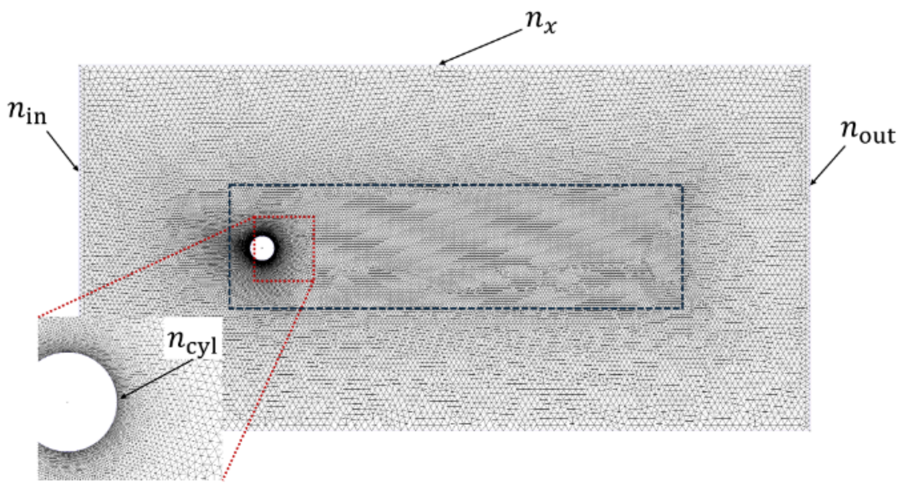

Figure 7: Schematic representation of the parametric mesh used in this study. Note the main number of elements for inlet (n_{in}), cylinder (n_{cyl}), outlet (n_{out}), top and bottom faces (n_x)). The dashed square indicates the area designed to improve the homogeneity of element sizes around the wake. There is a zoom over the cylinder to note the refinement done close to surface. The element distribution is uniform in spanwise and n_z is not displayed.

Mesh	L_z/D	n_{cyl}	n_x	n_{in}	n_{out}	n_z	Δz	$min(\Delta r)$	Elements	Elements/xy	$\overline{C_D}$	$\overline{C_{D_{RMS}}}$	$\overline{C_{L_{RMS}}}$
C	4	200	60	50	50	110	0,0364	0,0096	4,31E+06	3,92E+05	1,215	0,038	0,336
M1	4	240	70	50	50	130	0,0308	0,0050	5,29E+06	4,07E+05	1,233	0,040	0,347
M2	6	240	70	50	50	195	0,0308	0,0050	7,94E+06	4,07E+05	1,234	0,038	0,344
M3	12	240	70	50	50	360	0,0333	0,0050	1,45E+07	4,03E+05	1,233	0,035	0,342
F1	4	320	100	70	70	130	0,0308	0,0042	6,62E+06	5,09E+05	1,231	0,047	0,327
F2	4	380	100	70	70	150	0,0267	0,0025	7,94E+06	5,29E+05	1,236	0,041	0,380
F3	4	380	110	80	80	150	0,0267	0,0025	9,03E+06	6,02E+05	1,218	0,041	0,338

Table 1: Summary of grid independence study for $Re_D = 400$ of seven different meshes: coarse (C), medium (M) with different spanwise lengths, and fine (F1, F2, F3). All quantities are averaged over the last 200 time units.

2. Second, the manuscript lacks some essential discussions concerning the physical mechanism. The physical rationale behind the successful control relied much on the flow stability, especially the two distinct modes of 3D instability, which is also mentioned in the introduction part. However, the later discussions only focus on the instantaneous flow fields and statistical data, which are not enough to support any conclusion related to 3D stability.

We appreciate the reviewer's insightful comment regarding the need for a more thorough discussion of the physical mechanisms, particularly in relation to 3D stability. In response, we have added a detailed analysis of the three-dimensional flow dynamics using proper-orthogonal decomposition (POD). This new section explicitly examines the spatio-temporal characteristics of the flow structures in the uncontrolled, PC-, and DRL-controlled cases.

Our analysis reveals that the DRL control strategy effectively modifies the vortex-shedding frequency and alters the wake structure in a manner distinct from the PC control. Notably, the DRL-controlled flow exhibits more coherent structures and a larger recirculation bubble, indicating a significant impact on wake dynamics. Additionally, the POD analysis provides evidence of symmetry-breaking and three-dimensional transitions in the more energetic modes of the DRL-controlled case, supporting the notion that this control strategy influences 3D stability.

We believe that this addition addresses the reviewer's concern by providing a more in-depth discussion of the physical mechanisms governing the flow and their relation to stability.

Changes in the manuscript
In results section:

Additionally, we performed a proper-orthogonal-decomposition (POD) analysis \citep{lumley1967_POD} of the uncontrolled, PC and DRL-controlled cases at $Re_D=400$ to better understand their flow physics. The PODs were performed with the pyLOM package \citep{EIXIMENO2025109459} on the streamwise velocity and pressure fields over the last 40T_k, with a sampling rate of about 25 snapshots per vortex shedding.

Inspection of the frequency content of the temporal coefficients shown in Figure~\ref{fig:fig6_POD}(a) reveals that the DRL control acts selectively and less invasively. PC acts on a single frequency, resulting in a perturbation of the flow frequencies that can be seen in a more diffuse spectrum with a wider tail, thus indicating a stronger perturbation of the flow. In contrast, DRL control identifies and selects a wider range of frequencies to act on, resulting in a spectrum that is closer to that of the uncontrolled flow. As, a result, the flow exhibits a narrower range of frequencies yielding a less perturbed state.
%The modes of the DRL control are closer to those of the uncontrolled flow with a narrower bandwidth of active frequencies, whereas the modes of the PC case exhibit a wider range of frequencies, thus indicating a stronger perturbation of the flow.

Moreover, the streamwise component of the first POD mode is shown in Figure~\ref{fig:fig6_POD}(b) and the corresponding power-spectral densities are shown in Figure~\ref{fig:fig6_POD}(c). This mode contains a significant contribution of the vortex-shedding for the uncontrolled flow case. In the PC there is, in addition to the identified vortex shedding at $St\lvert_{\rm PC}=0.194$, as reported in Figure~\ref{fig:fig2}(c), the contribution of the actuation frequency at $f_c\lvert_{\rm PC}=0.171$. For the DRL-controlled flow we observe that the first mode closely resembles the control strategy found by the agent. Thus, DRL control is able to act on a wide range of frequencies, while in PC only acts at a single frequency. This effectively alters the vortex shedding frequency of the DRL-controlled flow to $St\lvert_{\rm{DRL}}=0.171$. In fact, DRL control actively works to modify the vortex shedding frequency from the uncontrolled state, $St\lvert_{\rm Uncontrolled}=0.2$. As a consequence, there are significant differences in the flow characteristics. In PC the resulting modes become highly disturbed on the far-wake, while the

near-wake remains mostly unaltered, exhibiting a strong similarity compared with the uncontrolled case. Furthermore, for the PC, it is observed that the double peak of $f_c \lvert_{\rm PC}$ and $St\lvert_{\rm PC}$ coexist, indicating that the PC is not successfully modifying the shedding in the same way as the DRL-controlled system does.

On the other hand, in the DRL-controlled flow the structures exhibit a more elongated wake structure and a larger recirculation bubble (measured from the end of the cylinder) of approximately $L_r/D = 2$, in contrast to the uncontrolled and PC recirculation bubbles of approximately $L_r/D = 0.8$ and $L_r/D = 0.9$, respectively. Subsequent modes exhibit more complex structures related to sub-harmonics and turbulent transition. The DRL-controlled flow is associated with modes that break the flow symmetries, even in the span-wise direction, suggesting a transition to three-dimensional flow at more energetic modes.

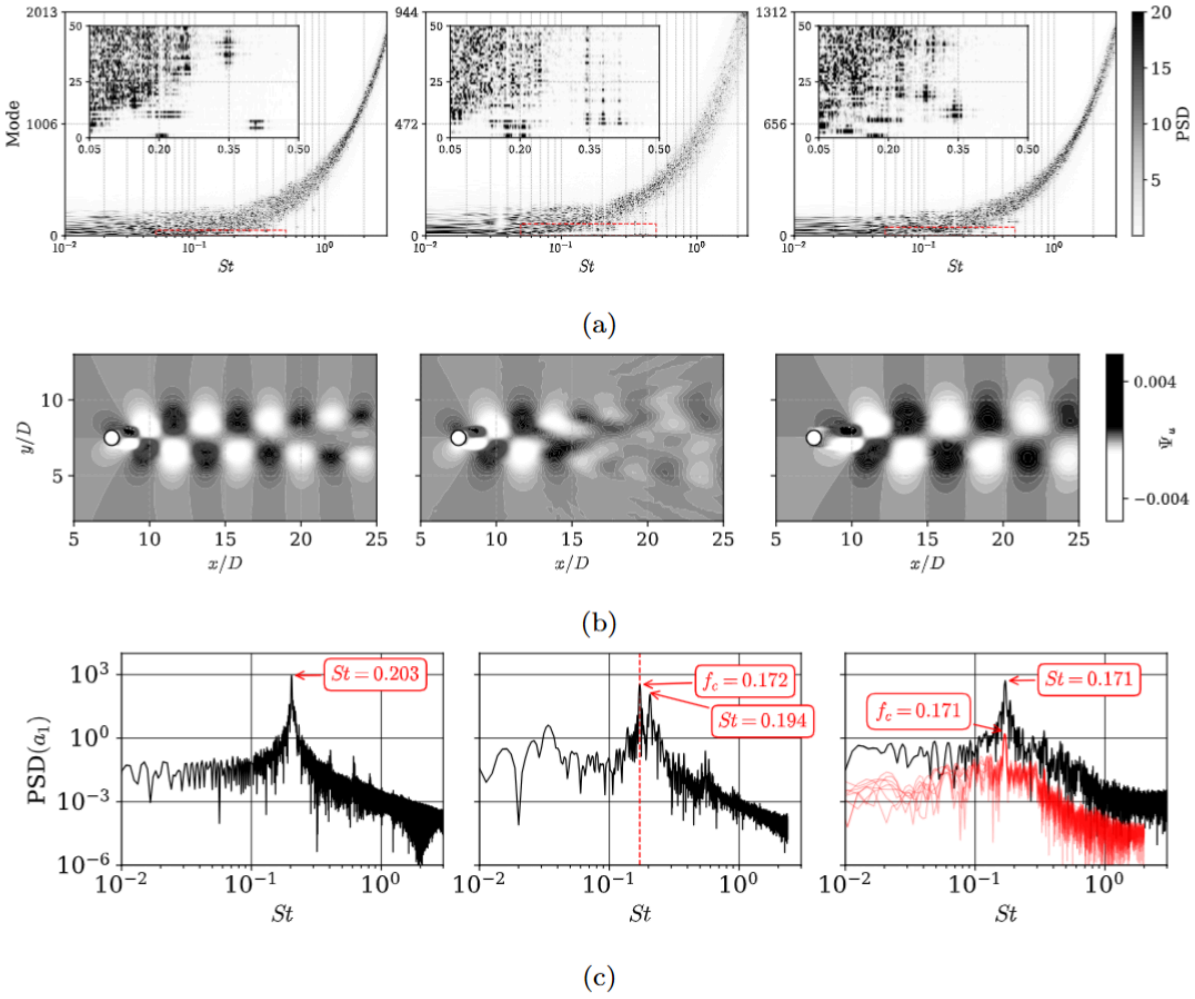

Figure 6: **Proper-orthogonal-decomposition (POD) results for streamwise velocity u at $Re_D = 400$ comparing, from left to right: uncontrolled, PC, and DRL-controlled case.** (a) Heatmap of the full spectrum for the POD modes and insert on top-left corner zooming into the first 50 modes within the dominant St. (b) Streamwise velocity for the first POD mode in the xy-plane (homogeneous in the spanwise direction). (c) Power-spectral densities corresponding to the first POD mode, highlighting the shedding Strouhal number (St) and the characteristic frequency (f_c), which appears as a double peak in the PC case. Note that for PC, a dashed red vertical line $f_c|_{\mathrm{PC}}$ is shown, while for DRL, the spectra of all Q_i are displayed in red with an offset to also highlight $f_c|_{\mathrm{DRL}}$.

In discussion section:

A POD of the DRL-controlled flow revealed the underlying physics of the actuation at $Re_D=400$, resulting in a stable wake structure with a longer recirculation bubble.

Specifically, I also have the following minor concerns:

3. In Figure 1, the periodic control is applied. It is questionable how these open-loop controls are found? Are they optimal? More details needed to be provided.

In order to answer this, we added Appendix 2, where details of how the optimization problem to find the best open-loop control is performed and how it becomes very difficult to assess due to the nature of this high-dimensional system to solve. We essentially sweep frequencies around the St and sweep Q values having the Qmax=0.176 as a reference, which coincides with the values that DRL framework is able to explore. Then we chose the best cases in order to compare in the results section. Heatmap tables are also presented for each Re_D case.

In addition, similar analyses have been performed after knowing the dominant frequencies and amplitudes of such a DRL strategy. Then we also have proved that those closed-loop strategies can not be reduced to a simple periodic control.

Changes in the manuscript

In results section:

For the study of the periodic control, more details are provided in Appendix 2, where the tables and heatmaps from the optimization problem are presented. These results will be used for the subsequent analysis.

Appendix 2 added:

This appendix aims to briefly introduce the study conducted to serve as a reference for a classical active control approach and be compared with the DRL performance. For this purpose, we opted for a periodic control, sweeping through frequencies \(f_c\) around the previously known \(St\) values for each case, and increasing \(Q\) up to \(Q_{\rm max}=0.176\) as a reference for maximum amplitude,

since this is allowed by the DRL framework for exploration. Thus, the actuation simplifies to a flow with a profile given by Equation(~\ref{eq:ujet}) in the xy-plane, extruded uniformly in the z-direction. In this case, however, $Q(f_c,t) = Q_{\rm max} \sin(2\pi f_c t)$.

Figure 8 shows the heatmaps of drag reduction for each of the Re_D values. It can be observed that it is difficult to identify a clear pattern, especially since we only have two degrees of freedom in this case. It appears that the system is highly sensitive to even small changes in f_c or $Q_{\rm max}$. The cases with the best drag reduction were selected for comparison in the study and serve as the reference for classic or periodic control.

Building on these results and the success of the DRL approach, we investigated whether the trained models could also be simplified using a spanwise extruded profile. In this approach, we can simply select the dominant $f_{c_{\rm DRL}}$ from the DRL actions spectra—refer to the table in Figure~\ref{fig:fig2}(c)—and perform a sweep around $Q_{\rm max}$ again, but now with values close to those obtained when running the deterministic cases—refer to Figure~\ref{fig:fig3_mfr}(a).

The results are enlightening: it is not possible to reduce the solution we already know from DRL to a simpler control. Achieving comparable drag reductions requires closed-loop control with the effective ability to transition to a stabilized control state. Furthermore, we can emphasize that beyond the transition, the exploitation of the three-dimensionalities becomes more necessary, and the performance of these simpler systems becomes ineffective.

$f_c\|_{PC}/St\|_{\text{Uncontrolled}}$		0,7	0,85	1	1,15	1,3
	$f_c\|_{PC}$	0,1148	0,1394	0,164	0,1886	0,2132
$Q_{\max}$	0,0088	-0,051	-0,463	0,557	19,010	-0,174
	0,0117	-0,217	-1,549	1,708	-0,051	-0,275
	0,0176	-0,825	-1,042	4,242	-0,101	-0,029
	0,0528	**-4,850**	4,365	15,825	15,825	-2,512

(a) $Re_D = 100$

$f_c\|_{PC}/St\|_{\text{Uncontrolled}}$		0,7	0,85	1	1,15	1,3
	$f_c\|_{PC}$	0,1302	0,1581	0,186	0,214	0,2418
$Q_{\max}$	0,0088	-0,900	-0,755	-1,919	0,191	-0,318
	0,0117	-0,306	-1,846	-0,100	1,574	0,701
	0,0176	-1,409	-3,956	1,785	2,163	0,410
	0,0528	**-8,322**	2,592	17,654	14,234	0,410

(b) $Re_D = 200$

$f_c\|_{PC}/St\|_{\text{Uncontrolled}}$		0,7	0,85	1	1,15	1,3
	$f_c\|_{PC}$	0,144	0,175	0,206	0,237	0,268
$Q_{\max}$	0,0088	-2,421	-0,908	5,900	-1,664	-1,740
	0,0117	-2,421	-3,933	8,926	-0,908	0,756
	0,0176	-4,690	**-6,959**	14,221	5,144	-1,664
	0,0528	-0,908	11,952	21,785	-0,151	-5,068

(c) $Re_D = 300$

$f_c\|_{PC}/St\|_{\text{Uncontrolled}}$		0,7	0,85	1	1,15	1,3
	$f_c\|_{PC}$	0,141	0,172	0,202	0,232	0,263132
$Q_{\max}$	0,0088	-2,526	-1,973	6,314	-6,077	-1,579
	0,0117	-4,499	**-9,629**	8,019	-5,525	-3,552
	0,0176	-7,751	-7,893	10,497	-5,975	-5,051
	0,0528	-0,710	18,564	37,332	10,576	-5,462

(d) $Re_D = 400$

Figure 9: Drag reduction percentage heat map (green is beneficial and red are degrading cases), $\Delta\overline{C_D}[\%] = 100 \times (\overline{C_D|_{\text{controlled}}}/\overline{C_D|_{\text{uncontrolled}}} - 1)$, summarizing the results of periodic control across a sweep of different frequencies, $f_c|_{PC}$, and amplitudes, $Q_{\max}$, for various Re_D. Values in bold represent the best cases selected for comparison against the DRL results.

$Q_{\max}$	0,0044	0,0059	0,0088	0,0117	0,0176	0,0528	0,0880	0,1232
$\Delta\overline{C_D}[\%]$	-0,101	-0,825	-1,549	-4,734	-1,339	11,235	19,314	20,530

(a) $Re_D = 100$ with control frequency set from known DRL, $f_c|_{\mathrm{DRL}} = 0.154$.

$Q_{\max}$	0,0044	0,0059	0,0088	0,0117	0,0176	0,0528	0,0880	0,1232
$\Delta\overline{C_D}[\%]$	-6,139	-8,191	-9,777	-6,139	-4,931	9,869	26,603	52,797

(b) $Re_D = 200$ with control frequency set from known DRL, $f_c|_{\mathrm{DRL}} = 0.173$.

$Q_{\max}$	0,0009	0,0018	0,0035	0,0044	0,0059	0,0088	0,0117	0,0176	0,0528
$\Delta\overline{C_D}[\%]$	-3,555	0,076	-1,740	-0,076	0,681	-1,362	-1,740	-5,673	4,0

(c) $Re_D = 300$ with control frequency set from known DRL, $f_c|_{\mathrm{DRL}} = 0.158$.

$Q_{\max}$	0,0009	0,0018	0,0035	0,0044	0,0059	0,0088	0,0117	0,0176	0.0528
$\Delta\overline{C_D}[\%]$	-0,868	-0,789	-2,605	-3,552	-3,315	-1,973	-5,028	-7,545	16,5

(d) $Re_D = 400$ with control frequency set from known DRL, $f_c|_{\mathrm{DRL}} = 0.171$.

Figure 10: Drag reduction percentage, $\Delta\overline{C_D}[\%]$, summarizing the results of periodic control across a sweep of different amplitudes, $Q_{\max}$. Note that this control is with fixed frequency taking the ones obtained from DRL, $f_c|_{\mathrm{DRL}}$, for each studied Re_D. Tables are color-coded to indicate performance: beneficial cases are shown in green, while degrading cases are shown in red.

4. Figure 1(a) seems to be not well converged.

Additional explanation has been added to justify the convergence of these training sessions. To summarize, in DRL, there is always necessary trade-offs about when to stop training in order to reach the best global maximum reward. The risk of stopping too early is also constrained by computational cost limitations and the size of the exploration space that the agent is able to explore.

Also, the colors and format of the figure 1(a) have been changed to be clearer and consistent with the manuscript.

Changes in the manuscript

In results section:

Hence, Figure~\ref{fig:fig1}(a) shows all the final rewards from the raw pseudo-environments, together with the pure drag reduction and lift-biased penalization contributions (see the Methods section for more details). As an example, the $Re_D=300$ scenario closely resembles the ideal training condition. This is because the curves exhibit minimal lift bias and result in a total reward that matches the pure drag reduction, stabilizing at R values that are manageable and simple to track. Similar patterns are obtained for the other Re_D cases indicating that the discovered policies are promising for all the cases. Note that we also observe several instances of apparent unlearning such as

for $Re_D=400$ at the episode 500 approximately. This is due to additional exploration of the agent, aimed at increasing the lift asymmetry (note the decrease of the blue line), but quickly returning to exploiting what the agent has identified as a well-performing policy. Once the reward value settles around a certain converged value of $R\approx 1.0$, the training process is concluded. \rev{The moment of stopping the training is always associated with a high-risk of both unlearning and having much more computational burden than expected. The best way to assess when training is concluded is to exploit the model with no exploration at different stages and observe any drop or improvement in performance.} Then, we proceed to the assessment of the learned policies in deterministic mode, which is discussed next.

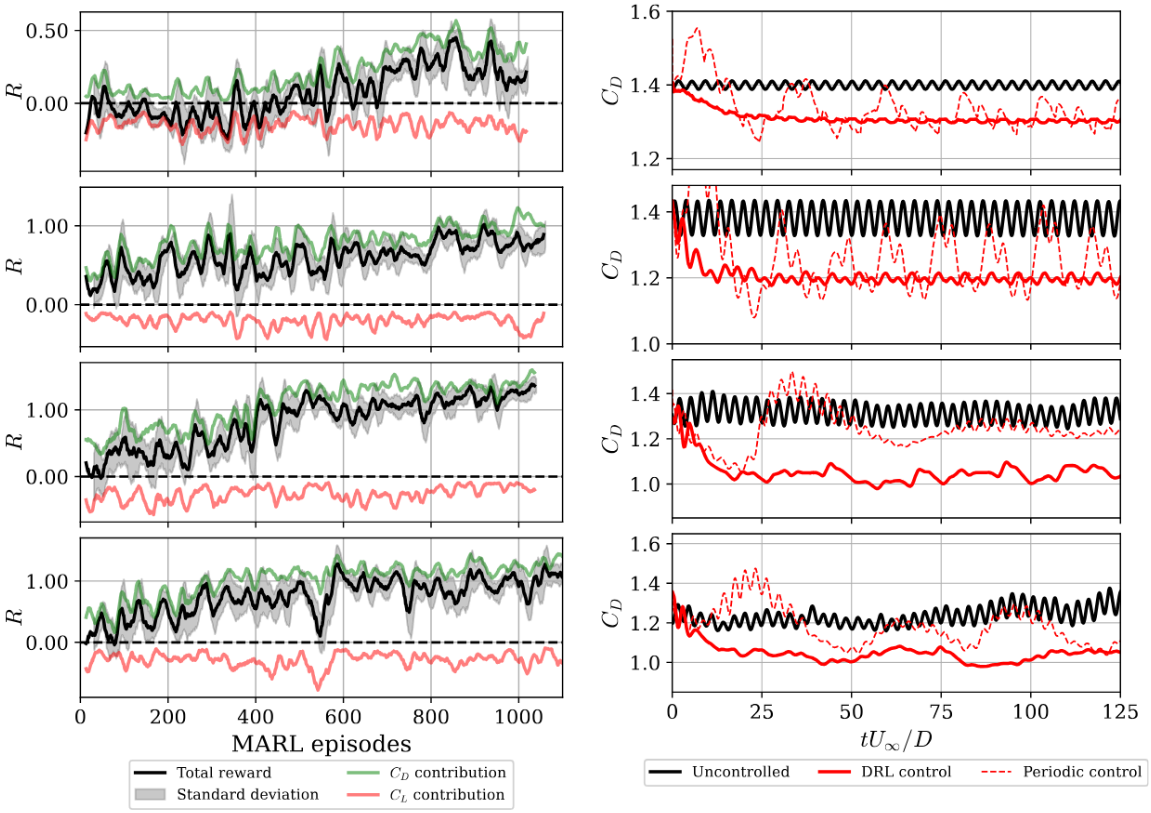

(a) (b)

Figure 1: **Reward evolution during exploration episodes and exploitation of the policies.** (a) Final total reward R together with its lift-bias and pure drag-reduction contributions during exploration in training sessions. Signals are smoothed by a moving average of 15 values, and the grey shaded area corresponds to the minimum and maximum rewards over those 15 episodes. (b) Drag-coefficient evolution during exploitation of the model. Comparison between uncontrolled, DRL control and PC. From top to bottom, $Re_D = 100$, 200, 300 and 400.

5. Eq.1 is used to quantify the flow rate of jet actuators, which practice is indeed common as a penalization term. However, can the authors also consider to quantify the energy consumption?

Energy consumption has been a topic of discussion for some time. To address this, we would need a reference mechanism, such as reservoirs or speaker membranes, that have already been measured for consumption at a given mass flow rate. However, this is not the case in our study. Instead, we propose using the mass-flow rate integral over time, compared with a reference value such as the crossflow mass intercepted by the entire cylinder. It is also useful to compare this with classical control methods that we studied. We observe that the DRL-based control actually oscillates around zero with very low amplitude, which provides a significant advantage over any constant flow setup. Some changes in the manuscript have been implemented to further clarify this point.

Changes in the manuscript

In results section next to Equation (1):

\rev{Note that while this evaluation of mass consumption is based on a numerical approach with modified Dirichlet boundary conditions, the actual mass cost would depend on the specifics of the experimental setup, including the type of actuators, such as membranes or reservoirs, and the jet configurations used. In the absence of experimental data for these components, we adopt this numerical approach. However, the present results indicate that the mass cost associated with DRL-based control remains minimal in comparison to the significant drag reduction achieved. This emphasizes the efficiency of DRL-based control strategies, as illustrated by the results in Figure~\ref{fig:fig2}, where the $E_{c}^*/\Delta \overline{C_D}$ ratio demonstrates that DRL requires only half the mass of classical control methods to achieve a comparable drag reduction.}

6. The spectra in Figure 2(b) are not well produced. The high-frequency region needs to be supplemented.

We appreciate the suggestion, and indeed, the y-axis has been extended to show this high-frequency region. However, for that coordinate in x/D and lower ReD, the PSD decreases rapidly once the first harmonics of the Strouhal number are surpassed. Also some minimum changes on nomenclature have been performed .

Changes in the manuscript

In results Figure 2(b):

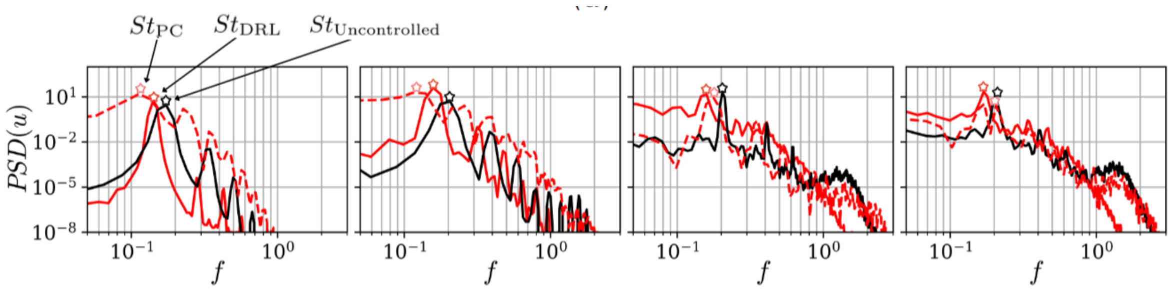

(b)

7. Figure 5(d, e) shows the velocity deficit, can the profiles be further related to drag reduction?

Yes, indeed, this decrease in deficit velocity is associated with a reduction in stresses and an expansion of the recirculation bubble. The distribution of momentum also changes, a fact that, according to wake theory for bluff bodies, makes perfect sense for drag-reduction mechanisms. Additional comment is added in the manuscript to expand this explanation.

Changes in the manuscript

In results:

Figures~\ref{fig:fig5_fields}(d) and \ref{fig:fig5_fields}(e) show how the wake changes noticeably, becoming slightly wider but decaying much faster as we move downstream. This is consistent with the observed decrease in deficit velocity, which is associated with a reduction in stresses and an expansion of the recirculation bubble. As the wake broadens, the distribution of momentum also shifts, which, according to wake theory for bluff bodies, is expected. Additionally, peaks in v fluctuations follow a similar pattern, indicating that the counter-rotating vortices are occurring further downstream as well. This expansion and faster decay of the wake, along with the altered vortex structure, provide insights into the changes in flow dynamics and support the idea that the wake evolution is strongly linked to the interplay between stress reduction and recirculation bubble growth.

8. In Page 23, a table that lists all the hyper-parameters is recommended.

We are thankful for the suggestion, and indeed, a table with the main hyperparameters used in the MARL framework has been added. This makes the manuscript more complete and will make it easier for readers to reproduce the results.

Changes in the manuscript In methodology:

**Parameter**	**Value**
n_{jets}	10
s_t size	297 (99 in 3 xy-slices)
s_t variable	Pressure
Q_{max}	0.176
Reward scalar K_R	5
Lift penalty α	0.6
Reward local weight β	0.8
Action duration $T_a[tU_\infty/D]$	0.25
Actions per episode	120
Time-smoothing function	Exponential
Batch size M	60
Epoch for optimizer	25
Strategy optimize policy	PPO
Architecture (networks)	2
Network 1 & 2 (type)	Fully connected layer
Network 1 & 2 (size)	512 neurons
Learning rate α_θ and α_ϕ	0.001
Likelihood ratio clipping ϵ	0.2
Discount factor γ	0.99
Optimizer type	Adam
Entropy regularization λ	0.01

Table 1: **Summary of the main parameters for the present DRL framework.**

We appreciate the thoughtful feedback from the reviewer, which has helped us to improve our work. In this rebuttal, we address the concerns raised, clarify ambiguities, and outline the steps taken to strengthen the manuscript. Our goal is to ensure that the revised paper meets standards of the journal and resolves all issues raised.
Each question is addressed in red, and any necessary changes to the manuscript are incorporated in blue.

Reviewer #2 (Remarks to the Author):

This paper utilize multi-agent reinforcement learning (MARL) to design active flow control strategies for the 3D flow around the cylinder. The flow control is considering multiple zero-net-mass-flux jet on top of the cylinder with the Reynolds number ranging from 100 to 400 covering the transition to 3D wake instabilities. This work is interesting and well-written, and it demonstrate that MARL can effective design an active flow control to achieve drag reduction and also show performance better than classical periodic control. Some comments below may help improve:

1. In figure 4, although the DRL is able to reduce the drag coefficient, the amplitude of lift coefficient is also reduced. This may be unfavorable in aerospace applications. Would the authors discuss this trade-off between drag coefficient and lift coefficient.

We acknowledge that the lift oscillations are reduced as a consequence of the drag reduction mechanisms employed by the DRL, which were not directly considered in the reward function of our approach. This trade-off between drag and lift is indeed an important aspect and presents an opportunity for future work. Specifically, tuning the reward function to include a more strict balance between drag and lift could be explored to better address this trade-off. In this study, however, our primary focus was on achieving pure drag reduction. We considered avoiding the risk of asymmetrical strategies with just a lift penalty, which would effectively force the lift signal to remain centered around zero. This approach could be investigated in subsequent iterations to refine and balance the aerodynamic performance further and maybe taking into account other quantities like maximizing lift RMS depending on the application, for example. In fact, the latter example could be explored by a different reward definition that look for maximizing the ratio to achieve better Cl/Cd or even taking into account root-mean-square values to control undesired oscillations.

Changes in the manuscript
In Results section:

We acknowledge that there is also a notable reduction in lift oscillations as a consequence of the drag reduction mechanisms employed by the DRL agents, which were not directly addressed in the reward r_t function. This trade-off is an important aspect and presents an opportunity for future approaches that may consider maximizing lift RMS or other metrics depending on the application.

2. Moreover, this work compared their flow control results with periodic control. However, how does this compared with other recent deep reinforcement learning results is suggested to be discussed. Is the authors using essentially the same reinforcement learning framework as others use, such as Ref [29], or the MARL in this paper has its own advantage?

In this case, the work done by Bae, J. & Koumoutsakos, P. (2022) shows a multi-agent RL implementation from the wall modelling perspective. Indeed the frameworks are similar conceptually but the application to an active flow control optimization problem makes it quite different. It would be interesting to see future reproducibility tests on different MARL implementations and also different architectures, for instance. One strong point of the present work is that the whole implementation is optimized to have good control authority over the bluff body and its quantities of interest, drag reduction in this case.

3. Page 23 the authors mentioned 'This algorithm demonstrates robustness, as it is somewhat forgiving with hyperparameter initializations'. I would be interested in seeing a robustness study regarding hyperparameter initialization.

We agree that a thoughtful study on this topic could lead to potential optimizations, particularly in the agent's hyperparameters. The intention of the quoted statement is to emphasize that, based on existing literature in RL with diverse applications and random seeds, PPO has demonstrated reliability and versatility for systems such as turbulent flows and other complex systems. We have revised the phrase to clarify this point.

Additionally, previous works by Rabault et al. (2019a) and Rabault et al. (2019b) have also explored the s_t iterations, among other aspects. In the present work, we mention how we trained multiple setups, varying parameters such as the neighboring observation state and actuator size. This process led to the best combination, which is the one reported here.

Changes in the manuscript
In results:

This algorithm demonstrates robustness, as it is relatively tolerant to initial hyperparameter settings and performs well across a wide variety of RL tasks without requiring extensive tuning. In this context, Ref.~\cite{rabault_accelerating_2019} is referenced, where the effects of parallel environments, action frequency update \(T_a/T_k\), and the smoothing law are studied. Also, Ref.~\cite{rabault_artificial_2019} where in Appendix E, different s_t had consistent results.

4. It is also suggested to discuss what is the challenge to apply this to higher Reynolds number turbulent flows.

We appreciate the suggestion and agree that applying this approach to higher Reynolds number turbulent flows poses significant challenges. As Reynolds numbers increase, the flow complexity requires finer resolution in both observation and control strategies, leading to higher computational costs. Additionally, more sophisticated reward functions and state representations may be necessary to address the broader range of turbulence scales effectively.

Notably, some follow-up work building on the present study has already explored these aspects, addressing this curiosity further. The results here set a new benchmark for the DRL community and may inspire applications to more turbulent cases or other Distributed Input-Distributed Output (DIDO) schemes.

For instance, recent work by Jeon et al. (2024) is interesting to explore where group invariants and positional encoding are exploited to improve the efficiency of training as a possibility to increase the Reynolds number.

Changes in the manuscript
In discussion:

This study sets a new benchmark for the DRL community, potentially inspiring its application to more complex turbulent scenarios at higher Reynolds numbers and to other Distributed Input-Distributed Output (DIDO) frameworks. Additionally, future research could improve training efficiency by implementing the approach in Ref.~\cite{jeon2024_invariants}, which explores the use of group invariants and positional encoding.

All the following typos have also been addressed.

Several Typos:

- On the top of page 5, 'The transion range of for 2D ... '
- Caption of figure 2, '(b) Power-spectral density of the streamwise velocity u evaluated at at the streamwise location... '
- Page 21, caption of figure 6, 'Note that is a schematic representation...' looks to miss the subject in this sentence
- Page 22, 'Drag and lift forces (Fx and Fy, respectively) forces are obtained ...'.

We appreciate the thoughtful feedback from the reviewer, which has helped us to improve our work. In this rebuttal, we address the concerns raised, clarify ambiguities, and outline the steps taken to strengthen the manuscript. Our goal is to ensure that the revised paper meets standards of the journal and resolves all issues raised.
Each question is addressed in red, and any necessary changes to the manuscript are incorporated in blue.

Reviewer #3 (Remarks to the Author):

The article presents the application of Deep Reinforcement Learning (DRL) to the flow around a circular cylinder at low Reynolds number and demonstrates that it can be used to reduce drag, via incorporation with an active flow control (AFC) mechanism. The work is ambitious and likely to be novel in a number of aspects. In the first case, although the Reynolds number range is low, it includes instances where the wake flow is three dimensional. Correspondingly, the AFC employed varies in the span wise direction. A considerable number of simulations were undertaken to train the model before the resulting framework was applied in an exploitation mode. In general I am in favour of this work being published, as it provides evidence for what should become an important branch of flow control in the coming years. However, there are a few issues to be discussed further, as noted below:

Conceptual

1. One of the main questions at present is whether or not the benefit of the multi agent vs single agent approach is fully demonstrated, ie MARL vs SARL. This is important as it is one of the main objectives and stated novelties of the work.

The advantages of multi-agent reinforcement learning (MARL) against single-agent reinforcement learning (SARL) have indeed been a focal point of this study, as it represents one of the core novelties of the work.

The primary advantage of MARL lies in its ability to exploit local invariants and deal with the curse of dimensionality, which speeds up the training process by simplifying the task each agent needs to learn. By distributing the input and output spaces among multiple agents, the overall exploration space becomes more manageable, making the training process feasible for high-dimensional problems such as this one. Computationally, this approach reduces the burden on individual agents and allows for more efficient learning and coordination among agents.

Additionally, we note that earlier studies using SARL were not successful in achieving the desired performance (considering computational cost). The inherent complexity of the problem, combined with the limitations of a single agent's ability to process global information effectively, proved to be a significant challenge. In contrast, the MARL framework demonstrated the capability to exploit localized information and coordinate actions across agents to achieve significant drag reduction.

Thus, MARL not only offers a practical solution to the computational challenges of training in high-dimensional spaces but also facilitates the emergence of effective control strategies that SARL approaches struggle to achieve.

This part of the Methods section has been rephrased and improved for clarity and enhanced understanding.

Changes in the manuscript

In methods section:

Previous work on 2D cylinders employed single-agent reinforcement learning (SARL) to implement various training stages. However, if the action space needs to handle multiple jets simultaneously---such as in the present 3D cylinder setup with distributed input forcing and a distributed output reward (the so-called DIDO scheme)---SARL is not viable. Unlike SARL, the multi-agent reinforcement learning (MARL) framework mitigates the curse of dimensionality by exploiting symmetries and training agents in local pseudo-environments. This approach makes high-dimensional control tasks more tractable by breaking them into smaller domains, allowing agents to maximize local rewards effectively. Furthermore, by distributing both the input and output spaces across multiple agents, the exploration space becomes significantly more manageable. This not only accelerates convergence during training but also ensures that the training process remains computationally feasible for such high-dimensional challenges.

It is important to note that earlier attempts using SARL, although not detailed in this study, were unsuccessful in achieving the desired performance—similar to the findings in Rayleigh-Bénard convection reported in Ref. \cite{effective}. In both cases, SARL struggled with handling global information effectively, leading to suboptimal control strategies. The limitations of the single-agent approach became evident even in simpler scenarios, where it failed to generalize across complex, high-dimensional environments. In contrast, MARL not only overcome these challenges but also demonstrated superior performance by leveraging localized coordination among agents. This ability to process distributed input and optimize distributed rewards makes MARL a much more effective framework, even in cases where SARL had previously been considered feasible.

2. Figure 3 provides an interesting view of the resulting mass flow across the jets from the exploitation phase of the DRL calculation. For Re=100 and 200, as stated by the authors, the flow is 2D and as such the resulting jet mass flow pattern is constant in spanwise as expected, but this raises two questions; 1) Is there a fundamental reason why the periodic control algorithm is not able to achieve the same performance? 2) would SARL have been able to obtain the same result and if so would it have been cheaper?

In response to the first question, the concept of exploiting invariants, even at low Reynolds numbers, allows the agent to explore beyond traditional or "extruded" control methods. In this case, while the agent has been exploring, it is clear that it does not find significant advantages in exploiting any three-dimensional effects–It is also interesting to observe how any trigger of three-dimensional effects is discarded. Additionally, an interesting feature to highlight is the two-stage actuation strategy: initially, an overshoot is required relatively quickly to decrease and stabilize the drag values later on. This type of behavior can be achieved with any basic periodic control method.

Furthermore, as demonstrated in the study introduced in the new Appendix 2, it is proven that this non-traditional closed-loop feedback is indeed necessary for optimal performance. This is consistent with the long-body DRL literature, dating back to the first work by Rabault et al. (2019).

Regarding the second question, as commented in Question 1, SARL was attempted, but due to computational constraints and the curse of dimensionality, the results were not satisfactory. In fact, to achieve the same number of explored trajectories, SARL would have been significantly more expensive than MARL. From our experience, it has also been observed that the activation of this control can occur at any moment, even during an uncontrolled state of the flow, with the agent consistently able to manage and stabilize it effectively.

Changes in the manuscript

In results section:

Although it might seem plausible that a single-agent SARL approach could achieve spanwise-uniform strategies due to its simplicity, computational limitations make this approach unfeasible. Specifically, SARL faces the curse of dimensionality, requiring exponentially more trajectories to explore the state space thoroughly. This results in a significantly higher computational cost compared to the multi-agent DRL approach, which is more efficient in navigating complex control strategies.

For the study of the periodic control, more details are also provided in Appendix 2, where the tables and heatmaps from the optimization problem are presented. These results will be used for the subsequent analysis.

Appendix 2 added:

Going beyond this, and with successful results from the DRL, we were also able to quickly verify whether these already trained models could be simplified in the same way, using a spanwise extruded profile. In this approach, we can simply select the dominant \(f_{c_{\rm DRL}}\) from the DRL actions spectra—refer to the table in Figure~\ref{fig:fig2}(c)—and perform a sweep around \(Q_{\rm max}\) again, but now with values close to those experienced when running the deterministic cases—refer to Figure~\ref{fig:fig3_mfr}(a).

The results are enlightening: it is not possible to reduce the solution we already know from DRL to a simpler control. Achieving comparable drag reductions requires that closed-loop control with the effective ability to transition to a stabilized control state. Furthermore, we can emphasize that beyond the transition, the exploitation of the three-dimensionalities becomes more necessary, and the performance of these simpler systems becomes ineffective.

$Q_{\max}$	0,0044	0,0059	0,0088	0,0117	0,0176	0,0528	0,0880	0,1232
$\Delta\overline{C_D}[\%]$	-0,101	-0,825	-1,549	-4,734	-1,339	11,235	19,314	20,530

(a) $Re_D = 100$ with control frequency set from known DRL, $f_c|_{\text{DRL}} = 0.154$.

$Q_{\max}$	0,0044	0,0059	0,0088	0,0117	0,0176	0,0528	0,0880	0,1232
$\Delta\overline{C_D}[\%]$	-6,139	-8,191	-9,777	-6,139	-4,931	9,869	26,603	52,797

(b) $Re_D = 200$ with control frequency set from known DRL, $f_c|_{\text{DRL}} = 0.173$.

$Q_{\max}$	0,0009	0,0018	0,0035	0,0044	0,0059	0,0088	0,0117	0,0176	0,0528
$\Delta\overline{C_D}[\%]$	-3,555	0,076	-1,740	-0,076	0,681	-1,362	-1,740	-5,673	4,0

(c) $Re_D = 300$ with control frequency set from known DRL, $f_c|_{\text{DRL}} = 0.158$.

$Q_{\max}$	0,0009	0,0018	0,0035	0,0044	0,0059	0,0088	0,0117	0,0176	0.0528
$\Delta\overline{C_D}[\%]$	-0,868	-0,789	-2,605	-3,552	-3,315	-1,973	-5,028	-7,545	16,5

(d) $Re_D = 400$ with control frequency set from known DRL, $f_c|_{\text{DRL}} = 0.171$.

Figure 10: Drag reduction percentage, $\Delta\overline{C_D}[\%]$, summarizing the results of periodic control across a sweep of different amplitudes, $Q_{\max}$. Note that this control is with fixed frequency taking the ones obtained from DRL, $f_c|_{\text{DRL}}$, for each studied Re_D. Tables are color-coded to indicate performance: beneficial cases are shown in green, while degrading cases are shown in red.

3. For Re=300 and 400 in fig 3, the variation of spanwise mass flow rate is shown, demonstrating the potential of the MARL via the independent actuation of the ten jets. However there are two further questions here: 1) there is a clear underlying frequency in both cases – how would a periodic control (PC) based on this frequency alone perform? Presumably SARL would be able to capture this frequency. 2) as such, in order to evaluate the net benefit of MARL over SARL, perhaps there should be a test of the significance of the spanwise variation over this baseline frequency. To what extent are DRL prescribed variations away from this frequency beneficial to the overall flow control?

In response to the first question, the results from the investigation, now reported in the new Appendix 2 and included in as a change in the manuscript in the previous question, show that it is not possible to replicate the performance of the MARL approach using a periodic control (PC) based on just the frequency and amplitude observed in the DRL deterministic results. The investigation demonstrates that the system requires closed-loop feedback in order to stabilize after a transient stage.

As for the second question, Appendix 2 includes a sweep of amplitudes (Qmax) while fixing the dominant frequency for each Reynolds number, further supporting the necessity of closed-loop feedback for effective flow control. This also demonstrates that at those Re exploiting the emergence of three-dimensionalities is needed and SARL, as discussed in previous questions, is not feasible.

A comment in the discussion section has been added to address this point.

Changes in the manuscript

In Discussion and conclusions section:

In fact, addressing the curiosity of whether these DRL results could be simplified or reduced to a basic control—one resembling a periodic control, but already knowing the dominant frequencies from the DRL that yield such good performance and range of Q—was investigated. The idea was perhaps to find strategies that do not require solving such a high-dimensional problem. However, the results reported in the second part of Appendix 2 indicate that achieving drag reduction is not feasible without considering the full spectrum of both temporal and spatial frequencies utilized by MARL agents. The results show that alternative approaches do not even achieve a quarter of the performance on average. This wider spectrum of closed-loop strategies is essential for addressing the three-dimensionalities and transitional stages required to shift from an uncontrolled to a stabilized controlled state.

4. The plots of Q with time in Figure 4a) and b) demonstrate the variation with time of mass flow rate. It is clear that there is relatively low spanwise variation of the jet mass flow, particularly once the ‘transition’ or adjustment period has been completed. Would a single jet (i.e. presumably via SARL) have obtained the same variation as that which is shown to be the predominant variation of Q? Did the authors explore the sensitivity of the jet width to the overall solution? E.g. starting with two jets per span and gradually increasing.

This is also addressed in the study presented in Appendix 2– as discussed in the previous question 3, where it is shown that a single jet (or extruded approach) is not successful. The size of the jet is primarily determined based on the hypothesis of having around two actuators in the spanwise direction per spanwise wavelength to effectively capture the three-dimensional structures. This configuration allows the agent to perceive and exert control over these structures, which are typically of the order of one diameter. However, attempts with slightly larger jets resulted in poorer performance, further supporting the idea that this specific configuration is optimal for effective control. Similar to what was discussed later in Question 10, a sensitivity analysis of this kind imposes a significant computational burden. We encourage future research to explore the optimal sizes and locations for these actuators as well.

Changes in the manuscript

In Methods section:

The cylinder has a total of two sets of $n_{{\rm jets}} = 10$ aligned synthetic jets that extend along its entire spanwise dimension, with a spanwise width $L_{{\rm jet}} = 0.4D$. This configuration enables having around two actuators per spanwise wavelength, which is known to be of the order of one diameter (mode-B).

5. Some of the conclusions made towards the end of the study, e.g. p19, first line, are around the performance of DRL vs PC strategies. Are authors confident, to a reasonable degree at least,

that there isn't a PC strategy that can achieve similar overall performance to the obtained DRL solution?

To address this question, we acknowledge the need for further explanation regarding the periodic control (PC) study, which was not fully detailed in the main body of the manuscript. To resolve this, we have reported this study for PC optimum frequencies in the new Appendix 2 as well. This appendix provides a comprehensive description of the optimization process used to identify the best open-loop control strategy.

The optimization involves sweeping across a range of frequencies around the Strouhal number (St) and amplitudes of Q, ensuring that the maximum Q matches the limits allowed in the DRL strategies. The best-performing cases from this analysis are selected for comparison in the results section. Heatmap tables summarizing the performance across different frequencies and amplitudes are presented for each Reynolds number (Re_D) case.

Additionally, a similar analysis has been conducted after identifying the dominant frequencies and amplitudes of the DRL strategies that contain richer spectrum of actuation. This further confirms that the closed-loop strategies obtained through DRL cannot be reduced to simple periodic control strategies. (As commented answering question 3)

Changes in the manuscript

In results section:
For the study of the periodic control, more details are also provided in Appendix 2, where the tables and heatmaps from the optimization problem are presented. These results will be used for the subsequent analysis.

In the Appendix 2 added:

This appendix aims to briefly introduce the study conducted to serve as a reference for a classical active control approach and be compared with the DRL performance. For this purpose, we opted for a periodic control, sweeping through frequencies \(f_c\) around the previously known \(St\) values for each case, and increasing \(Q\) up to \(Q_{\rm max}=0.176\) as a reference for maximum amplitude, since this is allowed by the DRL framework for exploration. Thus, the actuation simplifies to a flow with a profile given by Equation(~\ref{eq:ujet}) in the \(xy\)-plane, extruded uniformly in the \(z\)-direction. In this case, however, \(Q(f_c,t) = Q_{\rm max} \sin(2\pi f_c t)\).

Figure 8 shows the heatmaps of drag reduction for each of the \(Re_D\) values. It can be observed that it is difficult to identify a clear pattern, especially since we only have two degrees of freedom in this case. It appears that the system is highly sensitive to even small changes in \(f_c\) or \(Q_{\rm max}\). The cases with the best drag reduction were selected for comparison in the study and serve as the reference for classic or periodic control.

$f_c\|_{PC}/St\|_{\text{Uncontrolled}}$		0,7	0,85	1	1,15	1,3
$f_c\|_{PC}$		0,1148	0,1394	0,164	0,1886	0,2132
$Q_{\max}$	0,0088	-0,051	-0,463	0,557	19,010	-0,174
	0,0117	-0,217	-1,549	1,708	-0,051	-0,275
	0,0176	-0,825	-1,042	4,242	-0,101	-0,029
	0,0528	**-4,850**	4,365	15,825	15,825	-2,512

(a) $Re_D = 100$

$f_c\|_{PC}/St\|_{\text{Uncontrolled}}$		0,7	0,85	1	1,15	1,3
$f_c\|_{PC}$		0,1302	0,1581	0,186	0,214	0,2418
$Q_{\max}$	0,0088	-0,900	-0,755	-1,919	0,191	-0,318
	0,0117	-0,306	-1,846	-0,100	1,574	0,701
	0,0176	-1,409	-3,956	1,785	2,163	0,410
	0,0528	**-8,322**	2,592	17,654	14,234	0,410

(b) $Re_D = 200$

$f_c\|_{PC}/St\|_{\text{Uncontrolled}}$		0,7	0,85	1	1,15	1,3
$f_c\|_{PC}$		0,144	0,175	0,206	0,237	0,268
$Q_{\max}$	0,0088	-2,421	-0,908	5,900	-1,664	-1,740
	0,0117	-2,421	-3,933	8,926	-0,908	0,756
	0,0176	-4,690	**-6,959**	14,221	5,144	-1,664
	0,0528	-0,908	11,952	21,785	-0,151	-5,068

(c) $Re_D = 300$

$f_c\|_{PC}/St\|_{\text{Uncontrolled}}$		0,7	0,85	1	1,15	1,3
$f_c\|_{PC}$		0,141	0,172	0,202	0,232	0,263132
$Q_{\max}$	0,0088	-2,526	-1,973	6,314	-6,077	-1,579
	0,0117	-4,499	**-9,629**	8,019	-5,525	-3,552
	0,0176	-7,751	-7,893	10,497	-5,975	-5,051
	0,0528	-0,710	18,564	37,332	10,576	-5,462

(d) $Re_D = 400$

Figure 9: Drag reduction percentage heat map (green is beneficial and red are degrading cases), $\Delta\overline{C_D}[\%] = 100 \times (\overline{C_D|_{\text{controlled}}}/\overline{C_D|_{\text{uncontrolled}}} - 1)$, summarizing the results of periodic control across a sweep of different frequencies, $f_c|_{PC}$, and amplitudes, $Q_{\max}$, for various Re_D. Values in bold represent the best cases selected for comparison against the DRL results.

Flow Physics

6. At present, the emphasis of this work is placed more on the fact that the AFC is possible via the DRL framework employed, rather than a discussion of the flow physics of the identified DRL solution. Perhaps the latter could be explored further, particularly where it is relevant to the overall concept being demonstrated; i.e. that DRL results in non-intuitive solutions to be developed. This is important to the conclusion since as noted below there is only relatively minor spanwise variation of Q.

In response to the reviewer's comment, we have expanded the discussion of the flow physics in the DRL-identified solution by incorporating a proper-orthogonal-decomposition (POD) analysis. This analysis provides a clearer picture of how DRL influences the flow structures and frequency content compared to the uncontrolled and PC cases.

The results show that DRL modifies the vortex-shedding frequency while maintaining a more coherent wake structure with an elongated recirculation region. Unlike PC, which primarily adds perturbations at a fixed actuation frequency, DRL adapts across a broader frequency range. Additionally, higher-energy POD modes reveal symmetry-breaking structures in the DRL-controlled flow, suggesting the onset of three-dimensional transitions. These insights further support the idea that DRL identifies effective, non-intuitive control strategies with physically meaningful consequences.

This addition addresses the reviewer's concern by providing a more detailed examination of the DRL-controlled flow and its underlying mechanisms.

Changes in the manuscript

In the Results section:

Changes in the manuscript
In results section:

Additionally, we performed a proper-orthogonal-decomposition (POD) analysis \citep{lumley1967_POD} of the uncontrolled, PC and DRL-controlled cases at $Re_D=400$ to better understand their flow physics. The PODs were performed with the pyLOM package \citep{EIXIMENO2025109459} on the streamwise velocity and pressure fields over the last 40T_k, with a sampling rate of about 25 snapshots per vortex shedding.

Inspection of the frequency content of the temporal coefficients shown in Figure~\ref{fig:fig6_POD}(a) reveals that the DRL control acts selectively and less invasively. PC acts on a single frequency, resulting in a perturbation of the flow frequencies that can be seen in a more diffuse spectrum with a wider tail, thus indicating a stronger perturbation of the flow. In contrast, DRL control identifies and selects a wider range of frequencies to act on, resulting in a

spectrum that is closer to that of the uncontrolled flow. As, a result, the flow exhibits a narrower range of frequencies yielding a less perturbed state.
%The modes of the DRL control are closer to those of the uncontrolled flow with a narrower bandwidth of active frequencies, whereas the modes of the PC case exhibit a wider range of frequencies, thus indicating a stronger perturbation of the flow.

Moreover, the streamwise component of the first POD mode is shown in Figure~\ref{fig:fig6_POD}(b) and the corresponding power-spectral densities are shown in Figure~\ref{fig:fig6_POD}(c). This mode contains a significant contribution of the vortex-shedding for the uncontrolled flow case. In the PC there is, in addition to the identified vortex shedding at $St\lvert_{\rm PC}=0.194$, as reported in Figure~\ref{fig:fig2}(c), the contribution of the actuation frequency at $f_c\lvert_{\rm PC}=0.171$. For the DRL-controlled flow we observe that the first mode closely resembles the control strategy found by the agent. Thus, DRL control is able to act on a wide range of frequencies, while in PC only acts at a single frequency. This effectively alters the vortex shedding frequency of the DRL-controlled flow to $St\lvert_{\rm{DRL}}=0.171$. In fact, DRL control actively works to modify the vortex shedding frequency from the uncontrolled state, $St\lvert_{\rm Uncontrolled}=0.2$. As a consequence, there are significant differences in the flow characteristics. In PC the resulting modes become highly disturbed on the far-wake, while the near-wake remains mostly unaltered, exhibiting a strong similarity compared with the uncontrolled case. Furthermore, for the PC, it is observed that the double peak of $f_c \lvert_{\rm PC}$ and $St\lvert_{\rm PC}$ coexist, indicating that the PC is not successfully modifying the shedding in the same way as the DRL-controlled system does.

On the other hand, in the DRL-controlled flow the structures exhibit a more elongated wake structure and a larger recirculation bubble (measured from the end of the cylinder) of approximately $L_r/D = 2$, in contrast to the uncontrolled and PC recirculation bubbles of approximately $L_r/D = 0.8$ and $L_r/D = 0.9$, respectively. Subsequent modes exhibit more complex structures related to sub-harmonics and turbulent transition. The DRL-controlled flow is associated with modes that break the flow symmetries, even in the span-wise direction, suggesting a transition to three-dimensional flow at more energetic modes.

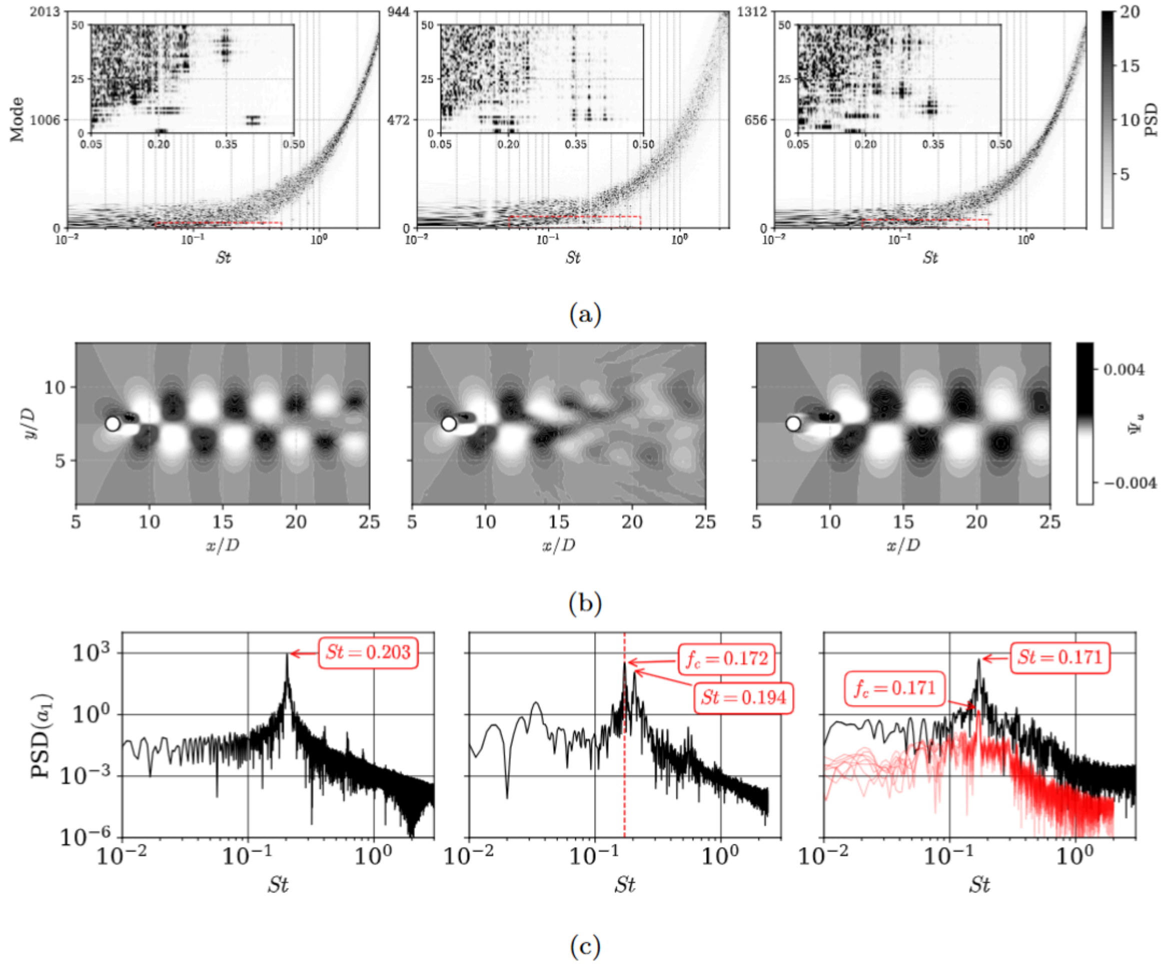

Figure 6: **Proper-orthogonal-decomposition (POD) results for streamwise velocity u at $Re_D = 400$ comparing, from left to right: uncontrolled, PC, and DRL-controlled case.** (a) Heatmap of the full spectrum for the POD modes and insert on top-left corner zooming into the first 50 modes within the dominant St. (b) Streamwise velocity for the first POD mode in the xy-plane (homogeneous in the spanwise direction). (c) Power-spectral densities corresponding to the first POD mode, highlighting the shedding Strouhal number (St) and the characteristic frequency (f_c), which appears as a double peak in the PC case. Note that for PC, a dashed red vertical line $f_c|_{\mathrm{PC}}$ is shown, while for DRL, the spectra of all Q_i are displayed in red with an offset to also highlight $f_c|_{\mathrm{DRL}}$.

In discussion section:

A POD of the DRL-controlled flow revealed the underlying physics of the actuation at $Re_D=400$, resulting in a stable wake structure with a longer recirculation bubble.

7. Could authors discuss the plateau observed in Cp for Re=400, as per figure 5f, at around theta=-90 degrees.

The plateau observed in the actuated case (red line) is primarily due to the small influence of the injected mass flow rates at this location. While this feature is noticeable, it does not significantly alter the overall pressure pattern or integral quantities, making its impact negligible when computing forces over the surface.

Additionally, we note that the figure now includes the ω marker and shaded area, indicating the location of the jets, which helps contextualize their small influence.

Changes in the manuscript

In the Results section:

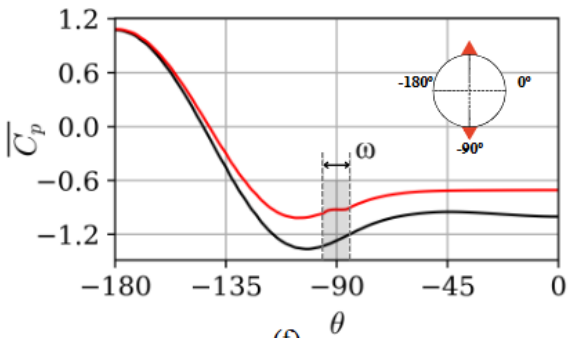

(f)

8. Could the authors provide indication of the boundary layer resolution and perhaps provide further insight on the impact of AFC on the location of boundary layer separation. This could be achieved via the analysis of velocity profiles in this region, and/or perhaps more succinctly via plotting of skin friction coefficient.

Thank you for your comment. The resolution of the boundary layer and the region close to the cylinder has been carefully addressed through grid independence and temporal convergence studies, which are detailed in Appendix 1. These studies ensure that the resolution is adequate for capturing the relevant flow structures near the wall.

Regarding the impact of AFC on boundary layer separation, it is important to note that accurately capturing boundary layer dynamics near the cylinder is challenging, particularly in the presence of the rapidly occurring separation around the cylinder, especially near the top and bottom of the wake (at approximately 90 degrees). Furthermore, the presence of the jets complicates this analysis.

Although we acknowledge the importance of this aspect, we focused primarily on the flow topology and wake dynamics in this study. Detailed analysis of boundary-layer behavior and its interaction with AFC is beyond the scope of this paper but would indeed provide valuable insights. This will be a topic for future investigations.

Changes in the manuscript:

Appendix 1 added:

A grid independence study was conducted to select an optimal mesh that balances low computational cost with accurate physical representation. This step is particularly important when working with the DRL framework due to its typically high CPU-hour demands and the necessity of parallelizing multiple simulations simultaneously. Striking the right balance is crucial.

The study involves iterating over a parametrized mesh, adjusting specific values to control the resolution in the \(xy\)-plane and the number of uniformly divided elements along the \(z\)-axis. Note that lengths $L_x/D=30$ and $L_y/D=15$ are the same for all cases. The schematic illustrates the placement of these elements and the parameters that can be adjusted. The table lists the details of each mesh—ranging from coarser to finer resolutions—and the resulting statistics for quantities of interest, such as \(\overline{C_D}\), \(\overline{C_{D_{\rm RMS}}}\), and \(\overline{C_{L_{\rm RMS}}}\), calculated over the entire surface. To ensure temporal convergence, 200 time units were found to be more than sufficient to achieve it for this Re_D range.

The results obtained for the drag coefficient (\(C_D\)) of a circular cylinder in the Reynolds number range of 100 to 400—\(C_D = 1.4\) at \(Re_D = 100\), \(1.38\) at \(Re_D = 200\), \(1.3\) at \(Re_D = 300\), and \(1.21\) at \(Re_D = 400\)—are in good agreement with the ranges reported in the literature (\(C_D \approx 1.2 - 1.4\) at \(Re_D = 100\), \(1.1 - 1.3\) at \(Re_D = 200\), \(1.0 - 1.2\) at \(Re_D = 300\), and \(1.0 - 1.2\) at \(Re_D = 400\)). A certain scatter in the data is observed, which can be attributed to the coexistence of two modes in this transition regime, as discussed in the works of \cite{tritton_1959, schlichting2016boundary, white2006viscous}. Regarding the Strouhal number St, the literature exhibited less scattering across \(Re_D\) and better alignment with the consensus in existing experimental and numerical literature. The first columns in the table in Figure~\ref{fig:fig2}(c) illustrate this agreement, comparing \(St\) values from the literature (Williamson, 1996 \cite{williamson_vortex_1996}) with the present statistics. Furthermore, the \(St\) values enable us to associate modes with our results as well.

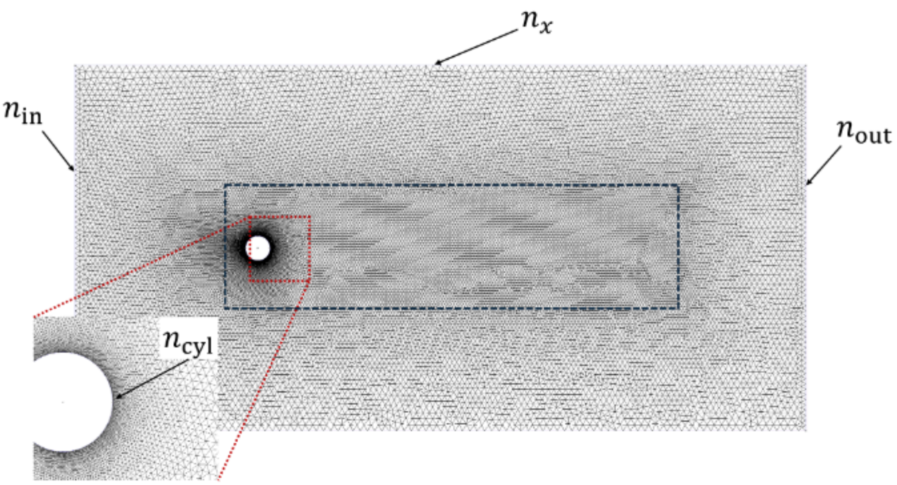

Figure 7: Schematic representation of the parametric mesh used in this study. Note the main number of elements for inlet (n_{in}), cylinder (n_{cyl}), outlet (n_{out}), top and bottom faces (n_x)). The dashed square indicates the area designed to improve the homogeneity of element sizes around the wake. There is a zoom over the cylinder to note the refinement done close to surface. The element distribution is uniform in spanwise and n_z is not displayed.

Mesh	L_z/D	n_{cyl}	n_x	n_{in}	n_{out}	n_z	Δz	$min(\Delta r)$	Elements	Elements/xy	$\overline{C_D}$	$\overline{C_{D_{RMS}}}$	$\overline{C_{L_{RMS}}}$
C	4	200	60	50	50	110	0,0364	0,0096	4,31E+06	3,92E+05	1,215	0,038	0,336
M1	4	240	70	50	50	130	0,0308	0,0050	5,29E+06	4,07E+05	1,233	0,040	0,347
M2	6	240	70	50	50	195	0,0308	0,0050	7,94E+06	4,07E+05	1,234	0,038	0,344
M3	12	240	70	50	50	360	0,0333	0,0050	1,45E+07	4,03E+05	1,233	0,035	0,342
F1	4	320	100	70	70	130	0,0308	0,0042	6,62E+06	5,09E+05	1,231	0,047	0,327
F2	4	380	100	70	70	150	0,0267	0,0025	7,94E+06	5,29E+05	1,236	0,041	0,380
F3	4	380	110	80	80	150	0,0267	0,0025	9,03E+06	6,02E+05	1,218	0,041	0,338

Table 1: Summary of grid independence study for $Re_D = 400$ of seven different meshes: coarse (C), medium (M) with different spanwise lengths, and fine (F1, F2, F3). All quantities are averaged over the last 200 time units.

9. As observed in Fig 4, there is an absence of unsteady flow structures in the immediate wake of the cylinder when the control is active; particularly in the region around 1 radius downstream of the aft of the cylinder. Could this be explored and explained in further detail?

This phenomenon is primarily related to the displacement of stresses caused by the stabilization of the flow. When the control is active, the stresses are moved downstream, reducing unsteadiness in this region.

It is important to note that drawing conclusions solely from instantaneous data can be risky, as such observations might not capture the overall statistical behavior of the system. This is why we further reinforce these findings using statistical data, as shown in subsequent figures. The statistical analysis confirms that the reduction in structures within the recirculation area is directly connected to the drag-reduction mechanisms, providing a more robust interpretation of the flow dynamics.

Furthermore, a note has been added to that part and figure 4 has been slightly updated in the revised manuscript to align with the new nomenclature and improve clarity for better understanding.

Changes in the manuscript

In Results section:

The observed reduction in unsteady structures within the recirculation bubble, or the increased absence of vortical activity in this region, can be associated with changes in the drag-reduction mechanisms. This interpretation is supported by statistical data presented later in this section in Figure~\ref{fig:fig5_fields}, which provides a more robust perspective on the flow dynamics.

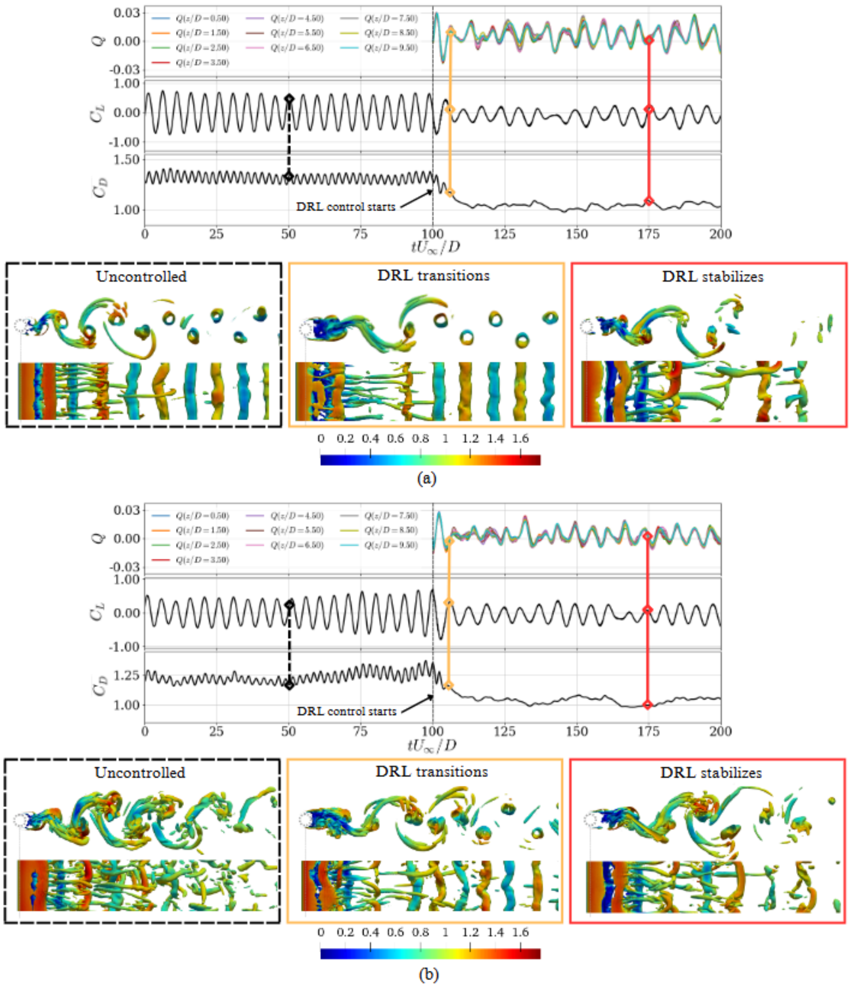

Figure 4: **Visualizations of the flow coherent structures during DRL-based strategy exploitation.** Temporal evolution of the flow at (a) $Re_D = 300$ and (b) $Re_D = 400$, from uncontrolled state to a stable DRL control. (Top) Non-dimensional mass-flow rate per unit width Q, lift coefficient C_L and drag coefficient C_D as a function of time; (bottom) snapshots showing vortical structures identified with the λ_2 criterion [35], where the isosurface $\lambda_2 D^2/U_\infty^2 = -0.5$ is shown for uncontrolled, transient, and DRL stabilized control states. The colors framing the flow visualizations correspond to the instants indicated in the temporal evolution of the relevant flow quantities.

Aspects of the DRL algorithm

10. What is the sensitivity of the algorithm to the location of observation states S_i? How does the location of the three slices arranged with respect to each individual jet – are they contained within the same spanwise extent? It is not clear.

First of all, it is important to note that the nomenclature has been changed in order to improve standardization with the existing literature. The observation state is now \(s_t \) instead of \(S_i \).

The observation states \(s_t \) were chosen to ensure that they capture critical flow dynamics while providing sufficient information for the agents' decision-making process. In fact, the sensitivity of the algorithm to the location and configuration of \(s_t \) was tested during the development of this setup. This included defining the length of the jet (\(L_{\text{jet}} \)), determining the size of the slices, and optimizing their placement in the spanwise direction. These tests were crucial, as this high-dimensional problem involves numerous degrees of freedom that need to be carefully managed to determine whether changes in performance are purely due to the location of the pressure probes.

In previous work, presented on ETMM proceeding conference paper (cited in the manuscript), a range of configurations was evaluated to analyze how the number of slices, their positioning, and the neighboring spanwise extent impacted the algorithm's ability to reduce drag effectively. The configuration presented in this study was selected as it consistently demonstrated better overall performance across all tested scenarios.

The following images show \(s_t \) was defined.

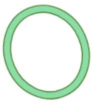

- Pressure distribution over cylinder surface

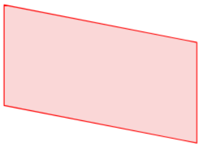

- Wake topology main features. Box length inspired from Moin 2000.

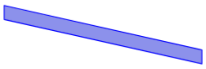

- Centerline with a bit more resolution: bubble length, symmetries, post processing, etc.

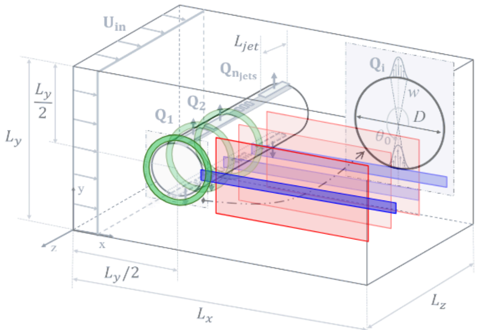

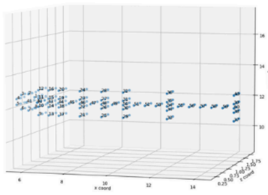

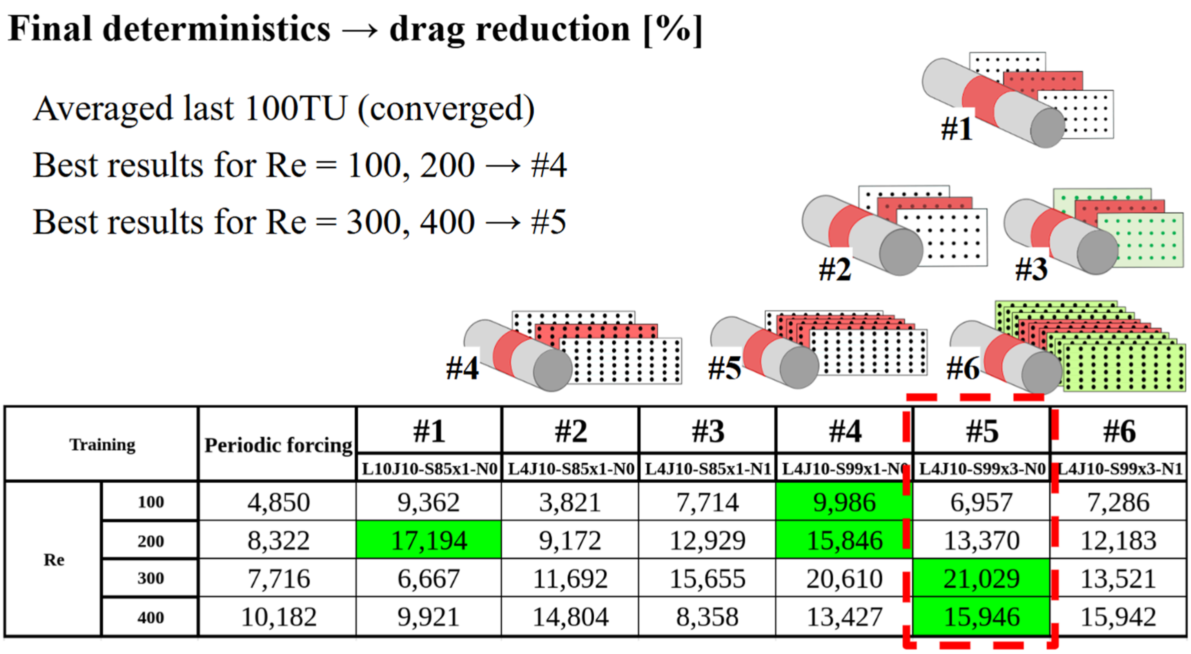

Final deterministics → drag reduction [%]

Averaged last 100TU (converged)

Best results for Re = 100, 200 → #4

Best results for Re = 300, 400 → #5

Training		Periodic forcing	#1<br>L10J10-S85x1-N0	#2<br>L4J10-S85x1-N0	#3<br>L4J10-S85x1-N1	#4<br>L4J10-S99x1-N0	#5<br>L4J10-S99x3-N0	#6<br>L4J10-S99x3-N1
Re	100	4,850	9,362	3,821	7,714	9,986	6,957	7,286
	200	8,322	17,194	9,172	12,929	15,846	13,370	12,183
	300	7,716	6,667	11,692	15,655	20,610	21,029	13,521
	400	10,182	9,921	14,804	8,358	13,427	15,946	15,942

We hope this explanation clarifies the rationale behind the current setup, as well as the steps taken to ensure that it was thoroughly tested and optimized. The following changes in the manuscript are implemented to comment on the sensitivity analysis:

Changes in the manuscript

In Methods section:

The observation state \(s_t \) provided to the agent consists of partial pressure values along the domain. This information is composed of three slices, each containing 99 pressure values, which are aligned with the corresponding jet in z/D coordinates and separated by $L_z/30D$. The probes or pressure values are concentrated in the wake and near-cylinder regions, allowing the agent to effectively exploit the spanwise pressure gradients.

In prior work in Ref.~\cite{suarez_2023_etmm}, various configurations were tested, including changes to the spanwise location and number of slices, to evaluate their impact on the performance of the drag reduction algorithm. These tests were crucial. The configuration chosen for this study was selected because it consistently provided the best overall performance across the evaluated scenarios.

11. What is the sensitivity to the quantity \Beta introduced at the top of p25? It appears that this quantity relates to the overall ability of the MARL to incorporate information from the non-local spanwise position. This would therefore appear to be a relevant parameter to explore in the context of the overall objectives and conclusions.

This parameter is important because it controls how much the MARL framework incorporates non-local spanwise information, which affects how well the agents coordinate. In the beginning, we found that if \(\beta \) is too small, the agents may focus too much on local information and struggle

to coordinate across the spanwise direction. On the other hand, if \(\beta \) is too large, the agents might overemphasize non-local information, adding potential noise to the signal and not really going towards the global goal.

We acknowledge that we did not test a wide range of \(\beta \) values, as this would have been computationally prohibitive given the complexity of the simulations. However, this limitation motivates future research to better understand the sensitivity of \(\beta \) and its role in balancing local and non-local information. Importantly, \(\beta \) plays a key role in facilitating coordination between agents, enabling neighboring agents to exchange meaningful information. This coordination is critical because what is beneficial for local performance may sometimes conflict with global objectives, and vice versa. By adjusting \beta=0.8, the framework seeks to strike a balance that harmonizes local and global priorities.

Although a more thorough exploration of \(\beta \) would provide valuable insights, our focus in this study was to demonstrate the feasibility of the MARL framework under practical conditions. We will add these points to the manuscript to highlight the importance of \(\beta \) and its potential implications for agent coordination and performance.

Changes in the manuscript

In Methods section:

This means that 80\% of the weight is assigned to the local value, while the remaining 20\% accounts for the global value. Based on our experience, the parameter \(\beta \) also acts as a smoother for the reward signal. If \(\beta \) is too high, the signal can become noisy, whereas a lower value significantly reduces fluctuations. Thus, in terms of learning a policy, the control authority, understood as the ability to influence the behavior of the system given feedback, can also be significantly influenced.

We acknowledge that we did not experimentally test a wide range of \(\beta \) values, as this would have been computationally prohibitive. However, this limitation motivates future research to better understand the sensitivity of \(\beta \) and its role in balancing local and non-local information. Importantly, \(\beta \) plays a key role in facilitating coordination between agents, enabling neighboring agents to exchange meaningful information. This coordination is critical because what is beneficial for local performance may sometimes conflict with global objectives, and vice versa. By defining \(\beta=0.8 \), the framework seeks to find a balance that harmonizes local and global priorities.

12. Can authors expand on the statements made on the comments regarding the limitation placed on the maximum values of Q in the algorithm employed. Specifically; p13 (4 lines from btm) 'less than 10% of the maximum possible value'; and p24 'Q_max=0.176 was set based on our experience…'. Why were these limitations necessary? Would it not be beneficial in some instances to permit larger active control?

This is an interesting discussion. The constraints were introduced for two main reasons:

Exploration and Learning Dynamics

Limiting Qmax ensures a balance between exploration and learning efficiency. If Qmax is too high, the exploration space becomes overly large, delaying convergence and potentially leading the agent to explore impractical or inefficient strategies. On the other hand, if Qmax is too small, the ability of the agent to explore meaningful dynamics is restricted, leading to a risk of having signal saturations (alternating -Qmax and Qmax). Based on experience and the results in this study, a value of 0.176 offered an effective middle ground, allowing sufficient exploration while maintaining stability.

Numerical Stability in CFD Simulations

Larger Qmax values can create boundary conditions that destabilize the CFD solver, requiring much smaller timesteps and complicating convergence. Keeping Qmax at 0.176 prevents this issue while still enabling meaningful control actions.

While allowing a larger control magnitude might seem beneficial in some cases, it introduces risks such as instability in learning, impractical control actions, and increased computational costs. The chosen limit ensures that the learned policy remains stable, efficient, and operationally feasible. We expand the discussion in the manuscript to clarify these points further.

Changes in the manuscript

In Methods section:

The DRL library outputs values in the range $a_t \in [-1, 1]$, requiring rescaling as $Q = a_t Q_{\rm{max}}$ to introduce the magnitude of actuation later in Equation~\ref{eq:ujet}. To balance exploration, learning efficiency and numerical stability, $Q_{\rm{max}} = 0.176$ was selected based on our experience with DRL for flow control. This value ensures meaningful exploration without introducing excessively large actuations that could destabilize the learning process or the CFD solver. If $Q_{\rm{max}}$ were too small, the exploration space would be overly constrained, potentially leading to premature policy saturation and suboptimal solutions. On the other hand, excessively large $Q_{\rm{max}}$ values could result in a very large exploration space that delays convergence or imposes boundary conditions that challenge the numerical stability of the simulation. Notably, $Q_{\rm{max}} = 0.176$ corresponds to twice the values used in the 2D cylinder setups~\cite{varela_deep_2022}, reflecting adjustments for the current configuration and objectives.

Numerical considerations

13. We presume that the code has been suitably validated for these flows, but it would be useful to confirm as such. It would also be helpful for repeatability to provide some details of spatial resolution employed, e.g. an image of mesh resolution to demonstrate where refinement has been prioritised.

This point has been addressed in the revised version by adding Appendix 1 already added as a change in previous question 8, which reports the grid independence study for Re_D = 400 as a reference. The quantities of interest were the convergence of C_d and RMS of C_d and C_l over time, which are in good agreement with the consensus in the extensive literature. Note that grid independence was also achieved for all Re_D cases, and the chosen mesh was M1 (medium), which offers a good balance between computational cost and accurately capturing the physics of the problem. This balance is crucial for any DRL training setup, given the high computational cost already associated with it. In the same Appendix 1, the parametrized mesh is also explained. Additionally, a comment in the results section referencing Appendix 1 has been added.

To further validate the findings, a column has been added to the table in Figure 2(c) to compare the Strouhal number with the literature which are very good agreement. In this case, the modes are also identified.

Changes in the manuscript

In Results section:

We then evaluate the optimal model and compare it with the uncontrolled cases and the periodic control (PC). Note that the uncontrolled converged results across Re_D are obtained after a grid-independence study—more details can be found in Appendix 1. For the study of the periodic control, more details are also provided in Appendix 2, where the tables and heatmaps from the optimization problem are presented. These results will be used for the subsequent analysis. In doing so, we investigate the utilization of the trained DRL agents for exploitation without involving any exploration. Statistical analyses are conducted to elucidate the underlying physical mechanisms responsible for drag reduction, leading to potential energy savings.

14. Consideration of figure 1b gives the impression that in some cases the flow has not fully developed, particularly for Re=300 and 400. Are authors confident that the DRL performance would remain similar for all stages of the flow. It is known from the literature that lower frequencies start to become more prominent – would the DRL be able to discern and adjust to counter these frequencies?

In Figure 1b, we originally displayed 100 time units, but we have now updated it and increased this time window to 125 time units to provide a clearer representation of the flow evolution. This change ensures that the flow's development is better captured. It is important to note that most simulations were run for 200 time units to ensure temporal convergence in all cases. Statistical quantities were computed within this time window, excluding the initial transients. The original choice to display only

100 time units was intentional, as it clearly highlighted the two-stage control strategy and supported the explanation in the manuscript.

For the actual quantitative assessment, we refer to the summary presented in Figure 2. While the appearance of low-frequency components can introduce scatter and complicate convergence, we observed robust performance, and temporal convergence after the activation of DRL control did not pose any issues. To mitigate risks associated with low frequencies, deterministic runs were conducted for at least three episodes. This also allowed us to assess whether the fixed episode duration posed any limitations.

Changes in the manuscript

In Results section:

The time window shown in Figure~\ref{fig:fig1}(b) is limited to 125 time units to better highlight the two-phase behavior of the DRL control. However, the actual simulations were run for at least 200 time units to ensure temporal convergence, with statistical quantities computed over the entire duration, excluding the initial transients.

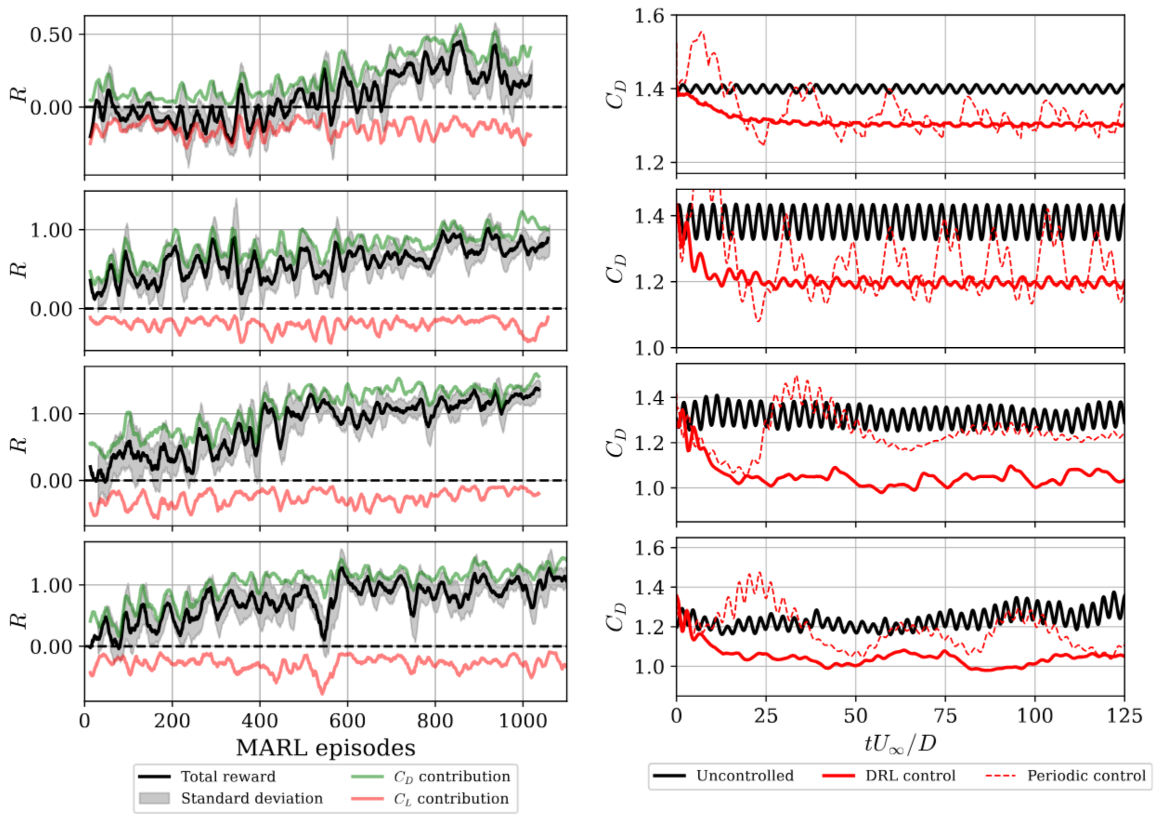

(a) (b)

Figure 1: **Reward evolution during exploration episodes and exploitation of the policies.** (a) Final total reward R together with its lift-bias and pure drag-reduction contributions during exploration in training sessions. Signals are smoothed by a moving average of 15 values, and the grey shaded area corresponds to the minimum and maximum rewards over those 15 episodes. (b) Drag-coefficient evolution during exploitation of the model. Comparison between uncontrolled, DRL control and PC. From top to bottom, $Re_D = 100$, 200, 300 and 400.

15. Some discrete behaviour in the solution is observed for the uncontrolled case in fig 5e e.g. x/D=9. Could the origin of this be explained?

We thank the Reviewer for pointing this out: it was indeed an error on the interpolation when creating the figure. The figure has been updated now.

Changes in the manuscript
In results section:

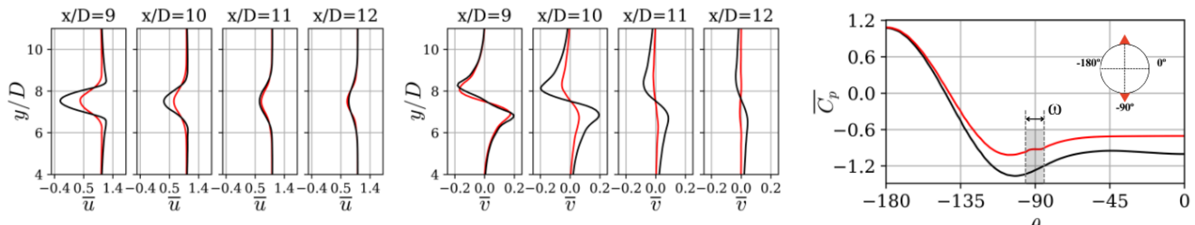

(d) (e) (f)

All the following typos and minor errors have also been corrected.

Typos/Minor:
p3 top: irruption -> eruption
p3 ‘table top games’ -> ‘board games’?
p15, fig 4. The line plots have x-axis label tU/D, while titles above flow vis. Frames have just t. Perhaps they should instead be t*=tU/D, or similar.
true! need to change those labels
P20: Are the two quantities ||U(Q, theta)|| in eqn (2) and ||U_jet||, defined three lines later, the same quantity? If so this should be clarified.
