# Supplementary material for: Flow control of three-dimensional cylinders transitioning to turbulence via multi-agent reinforcement learning
Source: Commun Eng. 2025 Jun 18;4:113. doi: 10.1038/s44172-025-00446-x (PMC12177081; doi:10.1038/s44172-025-00446-x)
Supplement: Supplementary file 2 — Transparent Peer Review file [file 44172_2025_446_MOESM2_ESM.pdf]

Supplementary Information

Flow control of three-dimensional cylinders transitioning to turbulence via multi-agent reinforcement learning

Pol Suárez[a], Francisco Alcántara-Ávila[a], Jean Rabault[b], Arnau Miró[c], Bernat Font[d], Oriol Lehmkuhl[c], Ricardo Vinuesa[a]

[a]*FLOW, Engineering Mechanics, KTH Royal Institute of Technology, Stockholm, Sweden*
[b]*Independent researcher, Oslo, Norway*
[c]*Barcelona Supercomputing Center, Barcelona, Spain*
[d]*Faculty of Mechanical Engineering, Technische Universiteit Delft, The Netherlands*

Supplementary information 1: Case validation and convergence

A grid-independence study was conducted to select an optimal mesh that balances low computational cost and accurate physical representation. This step is particularly important when working with the DRL framework due to its high computational demands and the necessity of parallelizing multiple simulations simultaneously. The study involves iterating over a parametrized mesh, refer to supplementary Figure 1, adjusting specific values to control the resolution in the xy-plane and the number of uniformly spaced elements along the z-axis. Note that lengths $L_x/D = 30$ and $L_y/D = 15$ are the same for all cases. The schematic illustrates the placement of these elements and the parameters that can be adjusted. The table lists the details of each mesh, ranging from coarse to finer resolutions, and the resulting statistics for the quantities of interest, such as $\overline{C_D}$, $\overline{C_{D_{\mathrm{RMS}}}}$, and $\overline{C_{L_{\mathrm{RMS}}}}$, calculated

Email addresses: `polsm@kth.se` (Pol Suárez), `rvinuesa@mech.kth.se` (Ricardo Vinuesa)

over the entire surface. To ensure temporal convergence, 200 time units were found to be more than sufficient to achieve it for this Re_D range.

The results obtained for the mean drag coefficient ($\overline{C_D}$) of a circular cylinder in the Reynolds-number range between 100 to 400—$\overline{C_D} = 1.4$ at $Re_D = 100$, 1.38 at $Re_D = 200$, 1.3 at $Re_D = 300$, and 1.21 at $Re_D = 400$—are in good agreement with the ranges reported in the literature ($\overline{C_D} \approx 1.2 - 1.4$ at $Re_D = 100$, $1.1 - 1.3$ at $Re_D = 200$, $1.0 - 1.2$ at $Re_D = 300$, and $1.0 - 1.2$ at $Re_D = 400$). All results are reported in supplementary Table 1. Some scatter in the data is observed, which can be attributed to the coexistence of two modes in this transition regime, as discussed in the works of [1, 2, 3]. Regarding the Strouhal number St, the literature exhibits less scattering across Re_D and better alignment with the reported values in the experimental and numerical literature. The first columns in the table in Figure 2(c) illustrate this agreement, comparing St values from the literature (Williamson, 1996 [4]) with the present statistics. Furthermore, the St values enable us to associate modes with our results as well.

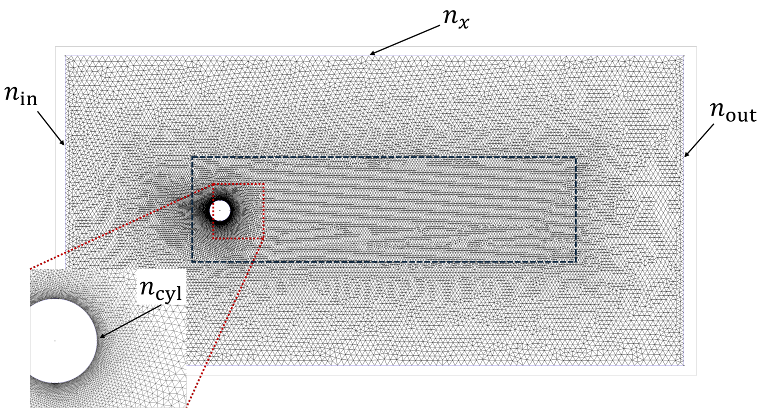

Supplementary Figure 1: Schematic representation of the parametric mesh used in this study. Note the main number of elements for inlet (n_{in}), cylinder (n_{cyl}), outlet (n_{out}), top and bottom faces (n_x)). The dashed square indicates the area designed to improve the homogeneity of element sizes around the wake. There is a zoom over the cylinder to note the refinement done close to surface. The element distribution is uniform in spanwise and n_z is not displayed.

Mesh	L_z/D	n_{cyl}	n_{x}	n_{in}	n_{out}	n_{z}	Δz	$min(\Delta r)$	Elements	Elements/xy	$\overline{C_D}$	$\overline{C_{D_{\text{RMS}}}}$	$\overline{C_{L_{\text{RMS}}}}$
C	4	200	60	50	50	110	0,0364	0,0096	4,31E+06	3,92E+05	1,215	0,038	0,336
M1	4	240	70	50	50	130	0,0308	0,0050	5,29E+06	4,07E+05	1,233	0,040	0,347
M2	6	240	70	50	50	195	0,0308	0,0050	7,94E+06	4,07E+05	1,234	0,038	0,344
M3	12	240	70	50	50	360	0,0333	0,0050	1,45E+07	4,03E+05	1,233	0,035	0,342
F1	4	320	100	70	70	130	0,0308	0,0042	6,62E+06	5,09E+05	1,231	0,047	0,327
F2	4	380	100	70	70	150	0,0267	0,0025	7,94E+06	5,29E+05	1,236	0,041	0,380
F3	4	380	110	80	80	150	0,0267	0,0025	9,03E+06	6,02E+05	1,218	0,041	0,338

Supplementary Table 1: Summary of grid independence study for $Re_D = 400$ of seven different meshes: coarse (C), medium (M) with different spanwise lengths, and fine (F1, F2, F3). All quantities are averaged over the last 200 time units.

Supplementary information 2: Periodic control study

This appendix aims to briefly introduce the study conducted to serve as a reference for a classical active control approach and be compared with the DRL performance. For this purpose, we opted for a periodic control (PC), sweeping through frequencies $f_c|_{\mathrm{PC}}$ around the previously known $St|_{\mathrm{Uncontrolled}}$ values for each case, and increasing Q up to $Q_{\max} = 0.176$ as a reference for maximum amplitude, since this is allowed by the DRL framework for exploration. Thus, the actuation simplifies to a flow with a profile given by Equation(2) in the xy-plane, extruded uniformly in the z-direction. In this case, however, $Q(f_c|_{\mathrm{PC}}, t) = Q_{\max} \sin(2\pi f_c|_{\mathrm{PC}} t)$.

Supplementary Figure 2 shows the heatmaps of drag reduction for each of the Re_D values. It can be observed that it is difficult to identify a clear pattern, especially since we only have two degrees of freedom in this case. It appears that the system is highly sensitive to even small changes in f_c or $Q_{\max}$. The cases with the best drag reduction were selected for comparison in the study and serve as the reference for classic or periodic control.

Building on these results and the success of the DRL approach, we investigated whether the trained models could also be simplified using a spanwise extruded profile. In this approach, we can simply select the dominant $f_c|_{\mathrm{DRL}}$ from the DRL actions spectra—refer to the Table 1 in the main manuscript, and perform a sweep around $Q_{\max}$ again, but now with values close to those obtained when running the deterministic cases—refer to Figure 3(a).

The results that are summarized in supplementary Figure 3 are enlightening: it is not possible to reduce the solution we already know from DRL to a simpler control. Achieving comparable drag reductions requires closed-loop control with the effective ability to transition to a stabilized control

state. Furthermore, we can emphasize that beyond the transition, the exploitation of the three-dimensionalities becomes more necessary, and the performance of these simpler systems becomes ineffective.

References

[1] D. J. Tritton, Experiments on the flow past a circular cylinder at low reynolds numbers, Journal of Fluid Mechanics 6 (1959) 547–567. doi:10.1017/S0022112059000829.

[2] H. Schlichting, K. Gersten, Boundary-layer theory, springer, 2016.

[3] F. M. White, Viscous Fluid Flow, 3rd ed., McGraw-Hill Education, New York, 2006.

[4] C. Williamson, Vortex Dynamics in the Cylinder Wake, Annual Review of Fluid Mechanics 28 (1996) 477–539.

$f_c\|_{\mathrm{PC}}/St\|_{\mathrm{Uncontrolled}}$		0,7	0,85	1	1,15	1,3
$f_c\|_{\mathrm{PC}}$		0,1148	0,1394	0,164	0,1886	0,2132
Q_{max}	0,0088	-0,051	-0,463	0,557	19,010	-0,174
	0,0117	-0,217	-1,549	1,708	-0,051	-0,275
	0,0176	-0,825	-1,042	4,242	-0,101	-0,029
	0,0528	**-4,850**	4,365	15,825	15,825	-2,512

(a) $Re_D = 100$

$f_c\|_{\mathrm{PC}}/St\|_{\mathrm{Uncontrolled}}$		0,7	0,85	1	1,15	1,3
$f_c\|_{\mathrm{PC}}$		0,1302	0,1581	0,186	0,214	0,2418
Q_{max}	0,0088	-0,900	-0,755	-1,919	0,191	-0,318
	0,0117	-0,306	-1,846	-0,100	1,574	0,701
	0,0176	-1,409	-3,956	1,785	2,163	0,410
	0,0528	**-8,322**	2,592	17,654	14,234	0,410

(b) $Re_D = 200$

$f_c\|_{\mathrm{PC}}/St\|_{\mathrm{Uncontrolled}}$		0,7	0,85	1	1,15	1,3
$f_c\|_{\mathrm{PC}}$		0,144	0,175	0,206	0,237	0,268
Q_{max}	0,0088	-2,421	-0,908	5,900	-1,664	-1,740
	0,0117	-2,421	-3,933	8,926	-0,908	0,756
	0,0176	-4,690	**-6,959**	14,221	5,144	-1,664
	0,0528	-0,908	11,952	21,785	-0,151	-5,068

(c) $Re_D = 300$

$f_c\|_{\mathrm{PC}}/St\|_{\mathrm{Uncontrolled}}$		0,7	0,85	1	1,15	1,3
$f_c\|_{\mathrm{PC}}$		0,141	0,172	0,202	0,232	0,263132
Q_{max}	0,0088	-2,526	-1,973	6,314	-6,077	-1,579
	0,0117	-4,499	**-9,629**	8,019	-5,525	-3,552
	0,0176	-7,751	-7,893	10,497	-5,975	-5,051
	0,0528	-0,710	18,564	37,332	10,576	-5,462

(d) $Re_D = 400$

Supplementary Figure 2: Drag reduction percentage heat map (green is beneficial and red are degrading cases), $\Delta\overline{C_D}[\%] = 100 \times (\overline{C_D|_{\mathrm{controlled}}}/\overline{C_D|_{\mathrm{uncontrolled}}} - 1)$, summarizing the results of periodic control across a sweep of different frequencies, $f_c|_{\mathrm{PC}}$, and amplitudes, Q_{max}, for various Re_D. Values in bold represent the best cases selected for comparison against the DRL results.

$Q_{\max}$	0,0044	0,0059	0,0088	0,0117	0,0176	0,0528	0,0880	0,1232
$\Delta\overline{C_D}[\%]$	-0,101	-0,825	-1,549	-4,734	-1,339	11,235	19,314	20,530

(a) $Re_D = 100$ with control frequency set from known DRL, $f_c|_{\mathrm{DRL}} = 0.154$.

$Q_{\max}$	0,0044	0,0059	0,0088	0,0117	0,0176	0,0528	0,0880	0,1232
$\Delta\overline{C_D}[\%]$	-6,139	-8,191	-9,777	-6,139	-4,931	9,869	26,603	52,797

(b) $Re_D = 200$ with control frequency set from known DRL, $f_c|_{\mathrm{DRL}} = 0.173$.

$Q_{\max}$	0,0009	0,0018	0,0035	0,0044	0,0059	0,0088	0,0117	0,0176	0,0528
$\Delta\overline{C_D}[\%]$	-3,555	0,076	-1,740	-0,076	0,681	-1,362	-1,740	-5,673	4,0

(c) $Re_D = 300$ with control frequency set from known DRL, $f_c|_{\mathrm{DRL}} = 0.158$.

$Q_{\max}$	0,0009	0,0018	0,0035	0,0044	0,0059	0,0088	0,0117	0,0176	0.0528
$\Delta\overline{C_D}[\%]$	-0,868	-0,789	-2,605	-3,552	-3,315	-1,973	-5,028	-7,545	16,5

(d) $Re_D = 400$ with control frequency set from known DRL, $f_c|_{\mathrm{DRL}} = 0.171$.

Supplementary Figure 3: Drag reduction percentage, $\Delta\overline{C_D}[\%]$, summarizing the results of periodic control across a sweep of different amplitudes, $Q_{\max}$. Note that this control is with fixed frequency taking the ones obtained from DRL, $f_c|_{\mathrm{DRL}}$, for each studied Re_D. Tables are color-coded to indicate performance: beneficial cases are shown in green, while degrading cases are shown in red.
